# Novel Intranasal Influenza-Vectored Vaccine Corfluvec Provides Protection Against Influenza and COVID-19, Mitigating SARS-CoV-2-Induced Lung Vascular Damage

**DOI:** 10.3390/vaccines14080684

**Published:** 2026-08-08

**Authors:** Marina Stukova, Anna-Polina Shurygina, Arman Muzhikyan, Ekaterina Romanovskaya-Romanko, Zhanna Buzitskaya, Marina Shuklina, Anastasia Pulkina, Daria Shamakova, Kirill Kryshen, Mariia Sergeeva, Dmitriy Lioznov

**Affiliations:** 1 Smorodintsev Research Institute of Influenza of the Ministry of Health of the Russian Federation, 197022 St. Petersburg, Russia; 2 RMC “Home of Pharmacy” JSC, 188663 Kuzmolovsky, Russia

**Keywords:** SARS-CoV-2, influenza, influenza-vectored vaccine, nucleocapsid protein, mucosal immunization, vascular and endothelial integrity, CD31, Ki-67, hamster, non-human primates

## Abstract

**Introduction**: The development of bivalent mucosal vaccines capable of providing protection against both influenza and SARS-CoV-2 is a major public health focus. While most COVID-19 vaccines target the spike (S) protein, the highly conserved nucleocapsid (N) protein represents a strategic target for cross-reactive, cell-mediated immunity. This study evaluates *Corfluvec*, an intranasal vaccine candidate based on an attenuated NS1-truncated influenza vector expressing a fragment of the SARS-CoV-2 N protein. **Methods**: Protective efficacy, including viral load and pathomorphological changes in the lungs and vessels, was evaluated in Syrian hamsters challenged with high and low doses of SARS-CoV-2 (lineage B.1.1). Cross-protective efficacy against homologous and heterologous influenza A strains (H1N1pdm09, H3N2, and A/PR/8/1934) was tested in a lethal murine model. Additionally, immunogenicity of *Corfluvec* applied via human-compatible delivery device was tested in cynomolgus macaques (*Macaca fascicularis*). **Results**: In Syrian hamsters, vaccination significantly reduced viral loads in the lungs and nasal turbinates. Histopathological analysis revealed a preservation of lung vascular integrity: vaccinated animals showed stable CD31 expression and controlled Ki-67 proliferative activity, accompanied by a marked reduction in vasculitis and perivascular edema compared to placebo controls. In mice, the vaccine provided 100% protection against homologous and heterologous influenza virus challenges. In macaques, the two-dose intranasal immunization was well-tolerated and induced significant systemic IgG and mucosal sIgA responses, alongside robust N-specific IFNγ+ T-cell activation. **Conclusions**: *Corfluvec* is a promising bivalent vaccine candidate that provides dual protection against influenza and COVID-19. Its ability to limit viral shedding from the upper respiratory tract and to mitigate SARS-CoV-2-induced pulmonary pathology, contributing to the preservation of lung vascular integrity, underscores the utility of mucosal immunization with *Corfluvec* as a valuable intranasal complement to current systemic vaccination strategies.

## 1. Introduction

Originating in China in December 2019, the COVID-19 outbreak led to the first coronavirus pandemic in human history. Its impact on human health, social stability, and economic development has been profound and far-reaching [1]. Large-scale vaccination campaigns—achieving coverage rates exceeding 70% in numerous countries—have significantly reduced COVID-19-associated morbidity and mortality. However, vaccination failed to fully prevent wave-like surges in COVID-19 incidence worldwide, which were associated with the emergence of antigenically distinct SARS-CoV-2 strains [2,3].

The vast majority of currently deployed vaccines are designed for intramuscular administration and aimed to induce systemic neutralizing antibodies against the full-length coronavirus Spike protein or its S1 subunit containing the receptor-binding domain (RBD) [4]. However, the S/RBD protein, which plays a critical role in viral attachment, fusion, and cell entry, undergoes the most pronounced antigenic changes due to increasing immune pressure driven by herd immunity [5]. Consequently, research into vaccines targeting conserved SARS-CoV-2 proteins is gaining increasing attention. Among these, the nucleocapsid protein (N) is a particularly promising target, as it is abundantly expressed during viral replication and plays a vital regulatory role across multiple stages of the coronavirus life cycle [6,7].

Although extensive fundamental research has established the central role of mucosal immunity in protection against antigenically variable respiratory pathogens, most COVID-19 vaccines are administered intramuscularly and primarily induce systemic immune responses. In contrast, mucosal immunity is crucial for preventing infection at the portal of entry [8], comprising innate immune factors, secretory immunoglobulin A (sIgA), and tissue-resident memory T (Trm) and B (Brm) cells, which serve as front-line defenders in the respiratory tract. TRM cells, in particular, are linked to long-term cross-protection against coronaviruses, including SARS-CoV-2 [9,10], while Brm cells support rapid local antibody responses upon re-exposure [11].

Intranasal immunization exploits the unique properties of the nasal mucosa: specialized M cells sample luminal antigens and transport them to NALT, where dendritic cells (DCs) process and present them to naïve T and B cells, driving their activation and differentiation [12]. This pathway establishes long-lived immune surveillance at the primary site of pathogen entry, enabling rapid local viral control and reducing transmission—a key advantage over systemic immunization [13].

To bridge this gap, the Smorodintsev Research Institute of Influenza in Russia has developed *Corfluvec*, a mucosal vector vaccine against COVID-19. Administered intranasally, this vaccine induces an immune response directly at the primary site of infection, thus limiting viral replication in the upper respiratory tract. By blocking infection at the early stages of viral exposure, it also helps prevent transmission to healthy individuals.

**Figure 1:**
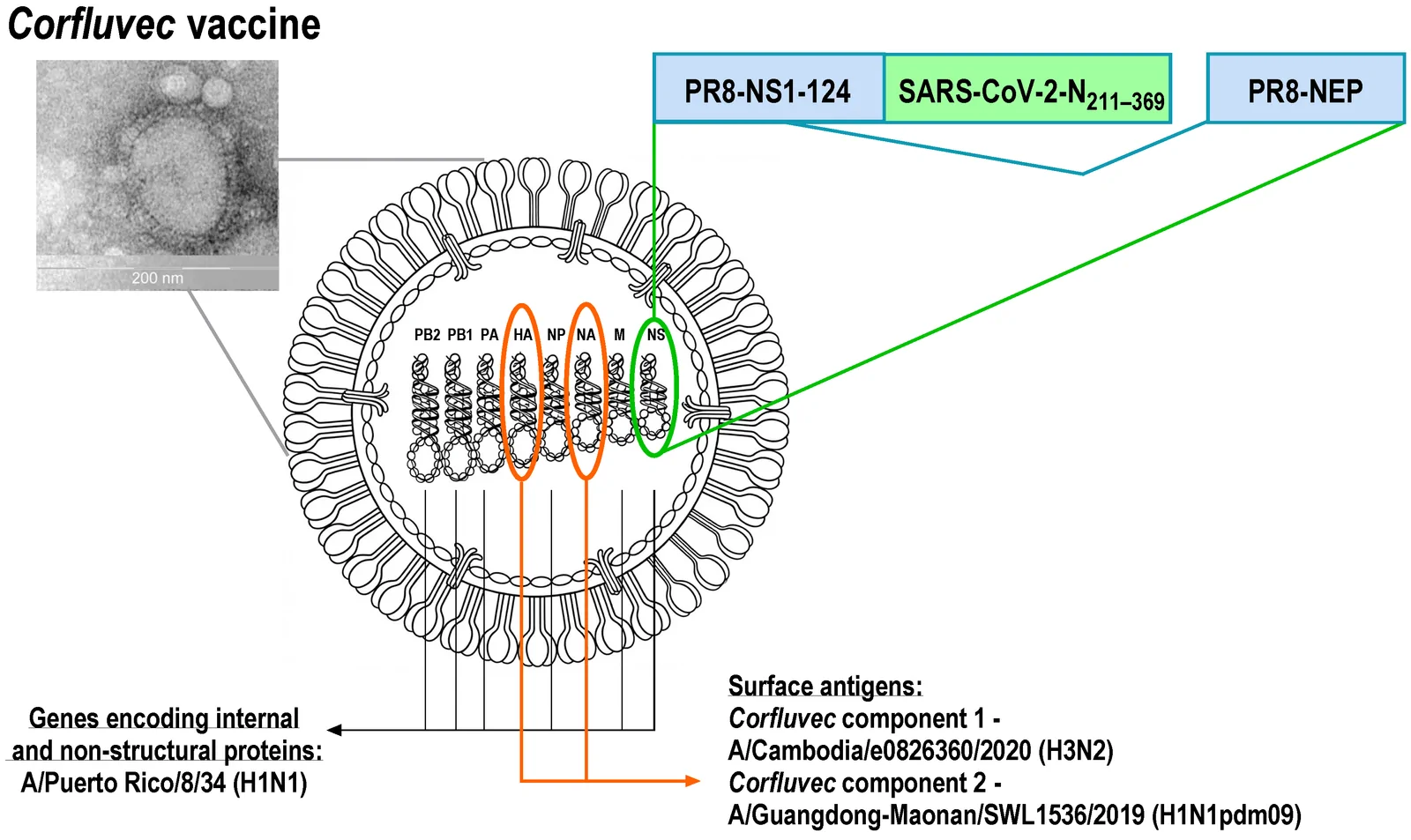
Schematic diagram of the *Corfluvec* vaccine strains. Vaccine strains have a classic 6:2 genome scheme including six genes from a highly productive donor A/PR/8/34 and two genes—hemagglutinin and neuraminidase—from influenza A viruses of epidemiologically relevant subtypes, A/Cambodia/e0826360/2020 (H3N2) or A/Guangdong-Maonan/SWL1536/2019 (H1N1pdm09). Virus attenuation was achieved by deleting the immunosuppressive domain of the NS1 protein (truncating it to 124 a.a.). A gene encoding the C-terminal fragment of the SARS-CoV-2 N protein (amino acids 211–369) from the B.1 lineage isolate was inserted in the open reading frame of the truncated NS1. The C-terminal region of the N protein was selected as the insertion antigen based on mapping data demonstrating a high density of potential T-cell and B-cell epitopes in this region.

The *Corfluvec* vaccine is based on an influenza A vector with a partially truncated NS1 gene encoding the C-terminal fragment of the SARS-CoV-2 N protein (aa 211–369), encompassing multiple predicted conserved T- and B-cell epitopes (Figure 1). Previously, we demonstrated that a two-dose intranasal immunization regimen with prototype vaccine strains FluVec-N (H1N1pdm09 and H1N1) protected naïve mice against heterologous SARS-CoV-2 challenge, despite the absence of detectable serum neutralizing antibodies [14].

In the present study, we extended these findings to antigenically updated H3N2 and H1N1 pdm09 vector strains—final *Corfluvec* vaccine formulation. The antigenically distinct strains were selected to enable a heterologous prime-boost strategy, which is designed to overcome potential vector-specific immune interference, particularly in influenza-pre-exposed populations. Furthermore, the use of antigenically distinct vectors broadens the anti-influenza immunity, potentially offering dual protection against both SARS-CoV-2 and multiple influenza subtypes. The relevance of influenza protection remains high, as seasonal and pandemic influenza continues to cause significant morbidity and mortality worldwide [15].

The *Corfluvec* vaccine strains exhibited essential production characteristics, including high growth in 10-day-old chicken embryos under standard vaccine manufacturing conditions (8.5 log_10_ TCID_50_/mL). They also displayed clear attenuation markers, both phenotypic (temperature-sensitive, ts phenotype) and genotypic (truncated NS1 reading frame), in compliance with the requirements for live vector vaccines. Here, we assessed protective efficacy—including vascular integrity—in the Syrian hamster model under varying immunization and challenge conditions, demonstrated broad protection against influenza in a murine model and evaluated immunogenicity in non-human primates (NHPs) using a human-compatible delivery device.

## 2. Materials and Methods

### 2.1. Vaccine

*Corfluvec* contains two recombinant vaccine strains, FluVec-N (H3N2, first component) and FluVec-N (H1N1pdm09, second component) (Figure 1), generated by reverse genetics using a previously described platform for creating attenuated influenza vectors with a modified NS genomic fragment containing foreign sequences [14]. The strains were produced by electroporation of Vero cells adapted to serum-free growth with a set of eight plasmids [16,17]. Subsequent passaging and amplification of the viruses were carried out in embryonated chicken eggs.

The vaccine was produced in embryonated chicken eggs. Purification involved tangential flow filtration (TFF), followed by diafiltration, resulting in a highly purified virus in a stabilizing buffer. The same stabilizing buffer was used as a placebo control in animal studies.

### 2.2. Animal Studies

All animals were housed under controlled conditions and monitored for any signs of disease. All experimental procedures were conducted in accordance with European and national regulations for the protection of experimental animals and were approved by the relevant local ethical committees. An overview of all animal studies conducted in this manuscript, including animal models, group sizes, vaccine strains, challenge strains, and immunization regimens, is provided in the Supplementary Materials (Table S1).

#### 2.2.1. SARS-CoV-2 Protective Efficacy Evaluation

**Figure 2:**
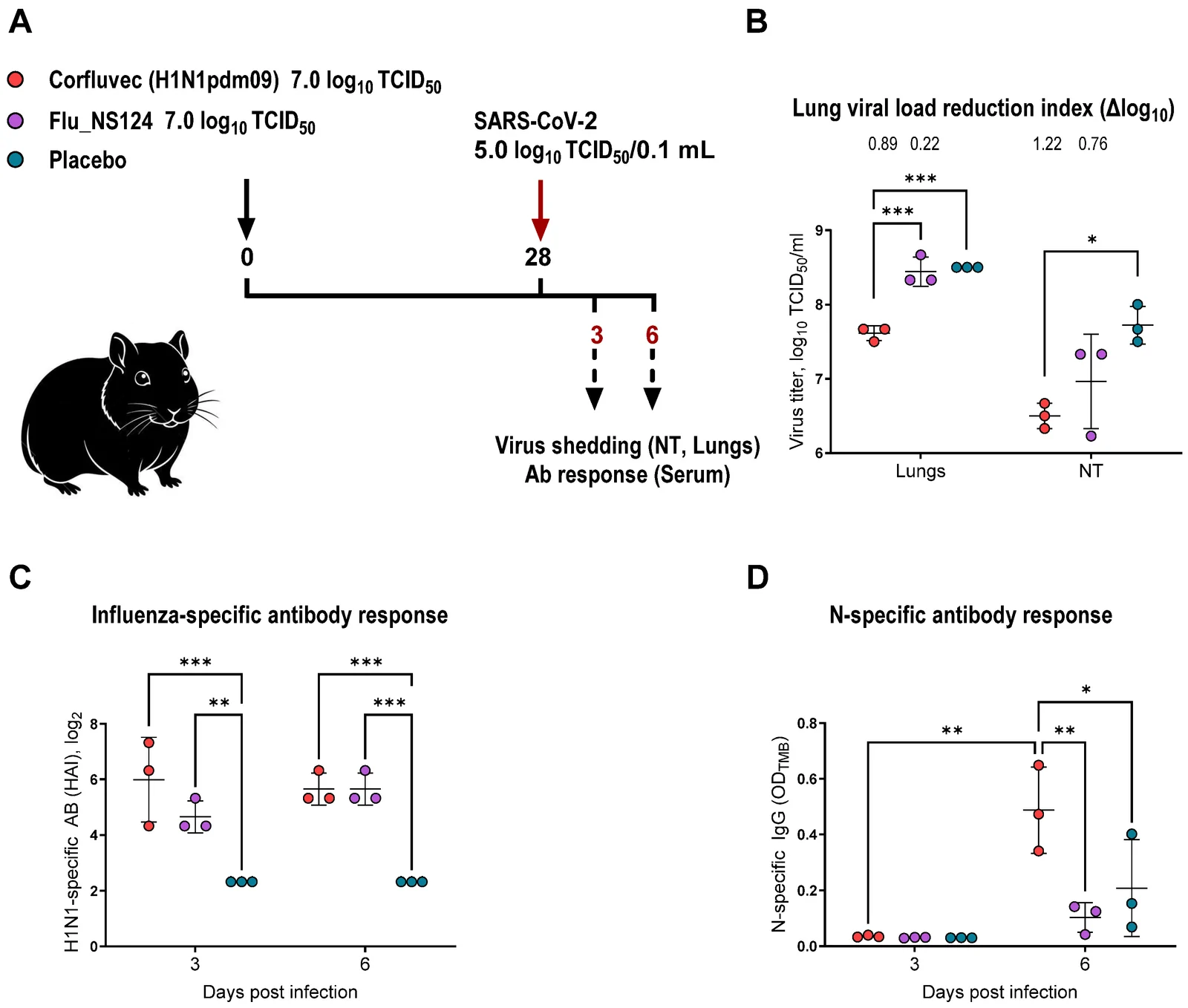
Protective effect of single immunization with FluVec-N (A/H1N1pdm09 vaccine component) and empty vector control Flu_NS124 in high-dose challenge experiment. (**A**) Study design. Male Syrian hamsters aged 3–4 months were immunized i.n. with 0.1 mL of vaccine strain FluVec-N (A/H1N1pdm09) (7.0 log_10_ TCID_50_/animal), empty influenza vector Flu_NS124 (7.0 log_10_ TCID_50_/animal) or Placebo. Infection of hamsters was carried out four weeks after immunization. Animals were i.n. infected with 5.0 log_10_ TCID_50_ of SARS-CoV-2 in 0.1 mL. Euthanasia was performed on day 3 (n = 3) and day 6 (n = 3) after infection. (**B**) Viral load in the respiratory tract organs (lungs, NT-nasal turbinates) on day 3 after SARS-CoV-2 infection. (**C**) H1N1-specific antibody response (HAI) in sera of hamsters on day 3 and day 6 after SARS-CoV-2 infection. (**D**) Antibody response (ELISA) to SARS-CoV-2 virus N-protein in sera of hamsters on day 3 and day 6 after SARS-CoV-2 infection. Data were considered statistically significant at *p* < 0.05 using one-way ANOVA (**B**) or two-way ANOVA (**C**,**D**) followed by Tukey’s multiple comparison test (*: *p* < 0.05, **: *p* < 0.01, ***: *p* < 0.001).

Male Syrian hamsters aged 3–4 weeks at the time of first immunization were used in this study (RMC “Home of Pharmacy” JSC, bioethics approval #2.4/21, 10 February 2021). Hamsters were allocated to groups using block randomization. In the preliminary single-dose efficacy experiment (Section 3.1.1), FluVec-N (A/H1N1pdm09) vaccine strain, was used in a dose 7.0 log_10_TCID_50_/0.1 mL/animal, under light ether anesthesia using a mechanical pipette 50 μL into each nostril (n = 6). Control animals were immunized either with empty vector Flu_NS124 (7.0 log_10_TCID_50_/0.1 mL/animal, n = 6) or with 0.1 mL of placebo (n = 6). Three weeks apart hamsters were challenged with SARS-CoV-2 (isolate 11308A, clade GR, lineage B.1.1) at a dose (5.0 log_10_ TCID_50_/0.1 mL/animal) (Figure 2A). In the second experiment (Section 3.1.2) hamsters were immunized with *Corfluvec* (7.0 log_10_TCID_50_/0.1 mL/animal, each component) or placebo twice i.n. at 3-week intervals (Figure 2A). Animals were monitored daily for clinical signs following immunization. Three weeks after the second dose, hamsters were challenged with SARS-CoV-2 (isolate 11308A, clade GR, lineage B.1.1) at either a high dose (4.5 log_10_ TCID_50_/0.026 mL/animal) or a low dose (3.0 log_10_ TCID_50_/0.026 mL/animal). Experimental groups included 11 animals each, with 5 animals in the intact (mock) group. Euthanasia was performed on days 3 (n = 4 per group), 6 (n = 4 per group), and 10 (n = 3 per group) post-infection.

The protective effect of the vaccine was evaluated based on clinical signs of SARS-CoV-2 infection, viral load in respiratory tract tissues (lungs and nasal passages), and lung pathomorphology, including assessment of pulmonary vascular damage and immunohistochemical (IHC) staining of lung sections for Ki-67 and CD31 markers.

For histological analysis, tissues were fixed in 10% neutral buffered formalin for at least 72 h and processed into paraffin blocks using standard procedures. Sections (5–7 μm) were stained with hematoxylin and eosin (H&E). Histological evaluation was performed using a Carl Zeiss AxioSkop 2 Plus light microscope (Carl Zeiss, Jena, Germany) at magnifications of 40×, 100×, 200×, and 400×. Microphotographs were acquired using an AxioCam ERc5s digital camera and AxioVision Rel. 4.8 software (Carl Zeiss, Germany). Morphometric measurements were carried out manually using AxioVision Rel. 4.8 (Carl Zeiss Microscopy GmbH, Jena, Germany) and the ImageJ software (version 1.53, National Institutes of Health, Bethesda, MD, USA). A semi-quantitative scoring system was used to assess lung pathology: 0—absent; 1—minimal damage; 2—mild; 3—moderate; 4—marked, and 5—severe. The percentage of affected tissue was estimated visually as the ratio of damaged area to the total section area.

For IHC analysis, sections were deparaffinized in three changes in xylene (10 min each), followed by rehydration through a graded ethanol series and washing in distilled water. IHC staining was performed using the immunoperoxidase method with the following primary antibodies: rabbit polyclonal anti-CD31 (Abcam plc, Cambridge, UK; catalog no. ab28364, 1:75 dilution), rabbit polyclonal anti-Ki-67 (Thermo Fisher Scientific, Waltham, MA, USA; catalog number PA5-19462, 1:100 dilution), and rabbit polyclonal anti-SARS-CoV-2 spike RBD domain (BIOCAD, St. Petersburg, Russia; series 06052020, 1:250 dilution). The workflow for identifying, imaging, and quantifying immunopositive areas is presented in Supplementary Materials (Figure S1A–E). Normal rabbit IgG was used as a negative control (Supplementary Materials, Figure S1F). A total of 45–50 microscopic fields per group were captured, and 2–3 tissue sections per animal were quantitative evaluated using image analysis software. The technical replicates were averaged to calculated the individual value for each animal. Morphometric analysis of SARS-CoV-2 antigen expression was carried out by quantifying the area of immunopositive staining per 1 mm of airway epithelium. Ki-67 expression was assessed in bronchial and bronchiolar epithelium and in vascular walls by counting positively stained nuclei per 100 μm tissue. CD31 expression in vascular walls was quantified as the area of immunopositive staining per 100 μm of endothelial surface.

#### 2.2.2. Influenza Protective Efficacy Evaluation

Outbred female mice (16–20 g, 6–8 weeks old) were obtained from the Rappolovo Laboratory Animal Breeding Facility (National Research Centre “Kurchatov Institute”, St. Petersburg, Russia). The experimental protocol was approved by the Bioethics Committee of the Smorodintsev Research Institute of Influenza (approval np. 45/1, 3 October 2021). Animals (n = 10 per group) were allocated to groups by block randomization and immunized i.n. with *Corfluvec* under light ether anesthesia (6.0 log_10_ TCID_50_/30 µL per dose, 15 μL into each nostril using a mechanical pipette), with a 21-day interval between immunizations. Control groups received placebo administered in the same manner.

Twenty-eight days after the second immunization, mice were challenged i.n. with wild-type influenza viruses (10 MLD_50_/30 μL per animal) using mouse-adapted strains: A/California/07/2009 (H1N1pdm09), A/Aichi/2/68 (H3N2), or A/PR/8/1934 (H1N1) obtained from the virus collection of the Smorodintsev Research Institute of Influenza (Ministry of Health of the Russian Federation). Body weight changes and survival were monitored for 9 days post-challenge.

#### 2.2.3. Immunogenicity in Non-Human Primates (NHPs)

Immunogenicity studies using human-compatible delivery device were conducted in *Macaca fascicularis* (RMC “Home of Pharmacy” JSC; bioethics approval #1.29/21, 21 June 2021). Four adult male macaques, aged 2.5–6 years, were immunized intranasally (i.n.) with 0.5 mL (0.25 mL per nostril) of each component of the *Corfluvec* vaccine (7.5 log_10_ TCID_50_ per animal), administered three weeks apart (Figure 10). Vaccination was performed using a syringe-based nasal sprayer approved for the delivery of live attenuated influenza vaccines (LAIV). The device consists of an insulin syringe fitted with a nozzle, enabling aerosol application of the formulation. One male and one female animal each received placebo (stabilizing buffer) following the same regimen. No randomisation was performed due to the small sample size; animals were allocated to experimental groups with consideration given to mean body weight. The a priori power analysis (two-sided *t*-test, α = 0.05, power = 80%) based on an expected very large effect size (Cohen’s d ≥ 2.0) derived from baseline comparisons, indicated that 4 animals were required for the internal repeated-measures evaluation (comparing post-immunization data with each animals’ own baseline parameters). Because of the high ethical and material costs associated with NHP research, the placebo group was minimized to 2 animals and served solely as a baseline environmental control rather than a fully powered comparative cohort.

Throughout the study, animals were monitored daily for clinical signs. Body weight and temperature were measured before the first immunization (day₋3) and on days 1, 5, 8, 21, and 42. To evaluate vaccine virus shedding, nasal swabs were collected from macaques on days 3 and 5 after immunizations and analyzed by RT-PCR. RNA was isolated from 100 µL of each sample using the RNeasy Mini Kit (Qiagen, Venlo, The Netherlands). Amplification was performed by real-time RT-PCR with the BioMaster RT-PCR-Extra reagent kit (Biolabmix LLC, Novosibirsk, Russia), with InfA-F (GACCRATCCTGTCACCTCTGAC) and InfA-R (AGGGCATTYTGGACAAAKCGTCTA) primers, and the InfA-P oligonucleotide probe (TGCAGTCCTCGCTCACTGGGCACG) recommended by the Centers for Disease Control and Prevention (CDC, Atlanta, GA, USA).

Humoral immune responses were evaluated prior to vaccination and on days 21 and 42 in serum samples (HAI, ELISA), bronchoalveolar lavage (BAL), and nasal washes (ELISA), as previously described [14,18].

To evaluate T-cell responses, peripheral blood mononuclear cells (PBMCs) were isolated from 5 mL of heparinized blood collected from macaques before each vaccination (day 0, day 21) and on days 7 and 20 after each vaccination (days 7, 27, 42). PBMCs were separated by density gradient centrifugation using 92% Ficoll solution (PanEco LLC, Moscow, Russia). Cells were cryopreserved in fetal bovine serum (FBS; Gibco, Thermo Fisher Scientific, Waltham, MA, USA) supplemented with 10% DMSO (AppliChem GmbH, Darmstadt, Germany)), using controlled-rate freezing to −70 °C (Cryo1 °C Freezing Container, “Mr. Frosty”, Thermo Fisher Scientific, USA), and were transferred to liquid nitrogen the following day.

After thawing, PBMCs were used in an IFNγ ELISPOT assay (Cellular Technology Limited, Cleveland, OH, USA) according to the manufacturer’s instructions. Cells were plated at 3.5 × 10^5^ cells/well and stimulated with either AIM V™ Medium AlbuMAX™ Supplemented (Gibco, Thermo Fisher Scientific, Waltham, MA, USA) in the presence of DMSO; PepTivator SARS-CoV-2 Prot_N (Miltenyi Biotec, Bergisch Gladbach, Germany; 5 µg/mL); H3N2 (A/Cambodia/e0826360/2020) or H1N1 (IVR-215 A/Victoria) monovalent split vaccine preparations, kindly provided by St. Petersburg Research Institute of Vaccines and Sera and the Enterprise for Bacterial Preparations of the Federal Medical-Biological Agency (SPbSRIVS FMBA, Krasnoe Selo, St. Petersburg, Russia; 5 µg/mL each). Stimulation with 0.05 µg/mL PHA with 1 µg/mL ionomycin (Sigma, Saint Louis, MO, USA) served as a positive control. Cells were incubated for 24 h and then analyzed using the ImmunoSpot S6 Ultimate UV Image Analyzer (CTL, USA) and ImmunoSpot Software (Cellular Technology Limited, Cleveland, OH, USA). Log_10_ fold changes (log_10_ FC) in spot-forming units per 3.5 × 10^5^ PBMCs were calculated after subtracting background values (medium-only) from antigen-stimulated counts.

### 2.3. Statistical Analysis

Data analysis in NHP and Syrian hamster experiments was performed in a blinded manner. During group allocation, conducted at RMC “Home of Pharmacy” JSC, the study director and the animal facility manager were aware of group assignments, as they were responsible for animal allocation/randomization and labeling. All samples submitted for analysis were transferred to the Smorodintsev Research Institute of Influenza under blind conditions. Personnel responsible for conducting the experiments, assessing outcomes, and per-forming primary data analysis was blinded to group allocation. The blinding code was not broken until all data collection and primary analyses were completed. No blinding procedures were applied in the mouse experiment. Data were analyzed using GraphPad Prism 10.4.0 (GraphPad Software, LLC, Boston, MA, USA). Results are presented as mean ± standard deviation (SD) or standard error of the mean (SEM). Statistical differences between groups were assessed using analysis of variance (ANOVA) or mixed-effects models, followed by multiple pairwise comparisons with appropriate *p*-value adjustment. Survival data were analyzed using the Mantel–Cox test. A *p*-value < 0.05 was considered statistically significant.

## 3. Results

### 3.1. Corfluvec Provides Dual Protection Against SARS-CoV-2 and Influenza

#### 3.1.1. Protective Efficacy Against SARS-CoV-2 in Syrian Hamsters

The protective efficacy of the candidate vaccine was assessed in two independent experiments using Syrian hamsters, a well-established model for SARS-CoV-2 infection that recapitulates key features of human COVID-19, including pulmonary pathology and vascular damage [19,20].

First, we assessed the activity of the FluVec-N (A/H1N1pdm09 vaccine component) following a single immunization against severe SARS-CoV-2 infection. The empty vector control (Flu_NS124) was included to distinguish between N-specific immunity and vector-mediated non-specific protection. Four weeks post-immunization, animals were challenged intranasally with a high dose of SARS-CoV-2 in large volume (5.0 log_10_ TCID_50_ in a volume of 100 μL) (Figure 2A).

By day 3 post-infection, control animals (Placebo group) exhibited high viral titers in the lungs (up to 8.5 log_10_ TCID_50_/mL), trachea (up to 6.7 log_10_ TCID_50_/mL), and nasal turbinates (up to 8.0 log_10_ TCID_50_/mL). In contrast, animals immunized with FluVec-N showed significant suppression of viral activity, with reductions of 0.9 log_10_ in the lungs and 1.2 log_10_ in the nasal turbinates (Figure 2B).

Notably, the Flu_NS124—empty influenza vector control group also showed a 0.76 log_10_ decrease in viral activity in the nasal turbinates. This effect is likely attributed to innate and trained immunity, a phenomenon previously described for live attenuated vaccines, including influenza, as well as recombinant influenza vectors with NS1-truncated protein [21,22,23,24].

Hemagglutination inhibition (HAI) assays on days 3 and 6 post-infection confirmed the presence of antibodies against the influenza A/H1N1 vector, indicating successful uptake of the vaccine virus in the nasal epithelium (Figure 2C). Serum antibodies targeting the SARS-CoV-2 N protein were detected on day 6 only in the FluVec-N group (Figure 2D). This confirms effective immune priming after a single intranasal dose and the subsequent mobilization of memory B cells upon viral challenge.

**Figure 3:**
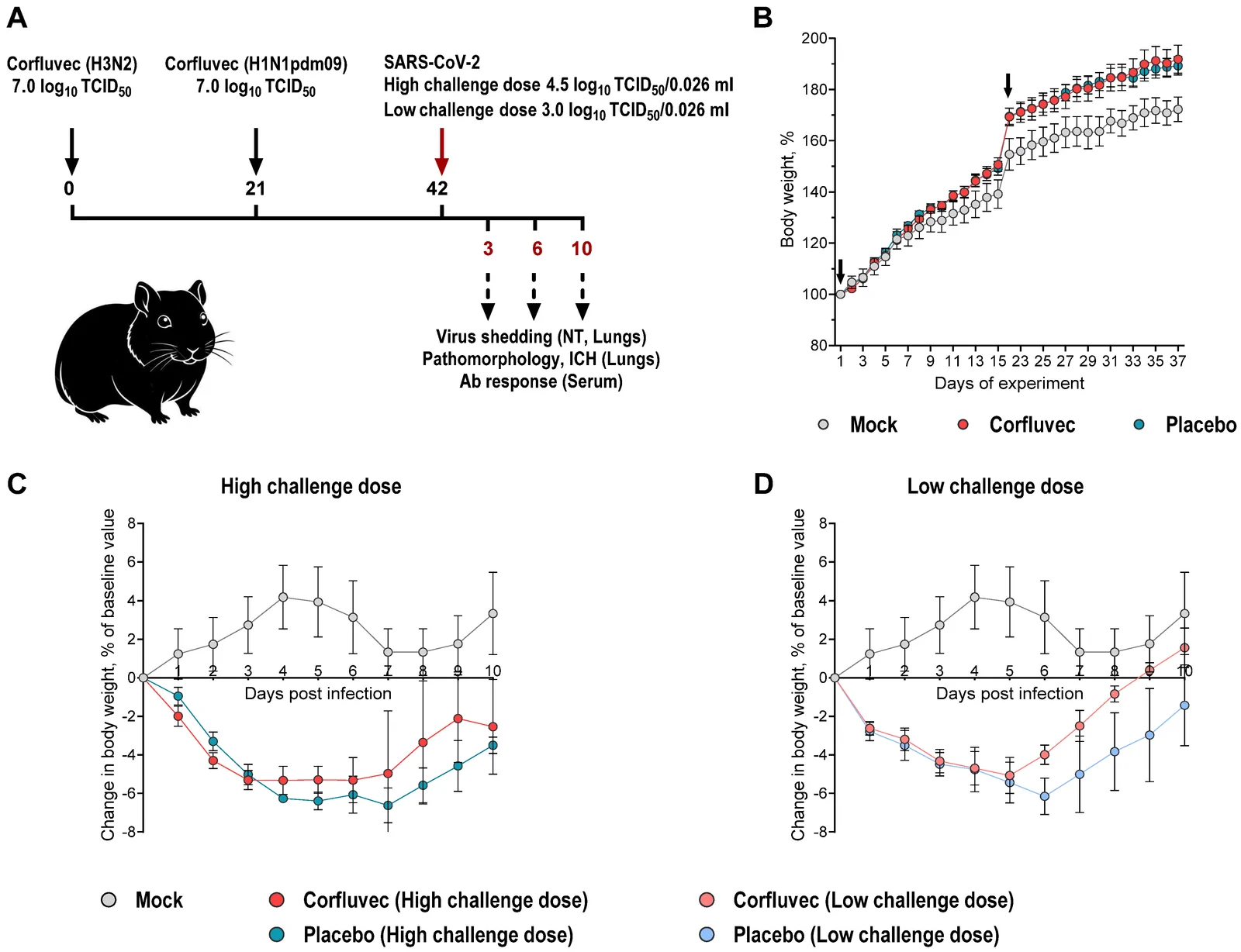
Protective efficacy of *Corfluvec* against SARS-CoV-2 in a Syrian hamster model—study design (**A**). Male Syrian hamsters aged 3–4 weeks were immunized i.n. with each component of the *Corfluvec* vaccine (7.0 log_10_ TCID_50_/animal in 0.1 mL) or Placebo, administered three weeks apart. Three weeks after the second immunization, animals were challenged with SARS-CoV-2. One cohort (n = 11 per group) received a high-dose challenge (4.5 log_10_ TCID_50_/animal), and the second cohort (n = 11 per group) received a low-dose challenge (3.0 log_10_ TCID_50_/animal). Euthanasia was performed on day 3 (n = 4), day 6 (n = 4), and day 10 (n = 3) post-infection. Protective efficacy was evaluated based on clinical signs of infection, viral load in respiratory tissues (lungs and nasal turbinates, NT), and histopathological changes in the lungs, including immunohistochemistry (IHC) analysis. Body weight dynamics (% of baseline) following immunization (**B**). Black arrows indicate the first and second immunisations. Body weight changes after SARS-CoV-2 infection. Mean relative body weight (% of baseline) is shown for immunised and control animals following high-dose (**C**) and low-dose (**D**) challenge (mean ± SEM).

In this experiment, despite a significant reduction in viral load in FluVec-N vaccinated animals, pathomorphological analysis revealed substantial lung tissue damage in all groups (Supplementary Materials, Figure S2). This is likely attributable to the intrapulmonary administration of a high dose of challenge virus in a large volume (100 µL). Therefore, for subsequent experiments evaluating protective efficacy of *Corfluvec*, we used two challenge doses of SARS-CoV-2- low (3.0 log_10_ TCID_50_/animal) and high (4.5 log_10_ TCID_50_/animal), which were administered in a smaller volume of 26 µL (Figure 3A). This volume was selected as the minimum required to deliver the target challenge dose (4.5 log_10_ TCID_50_/animal) while minimizing non-specific tissue damage, consistent with observations that reducing inoculum volume decreases non-specific pulmonary pathology [25,26].

Immunization with *Corfluvec* had no adverse effects on general condition or body weight (Figure 3B). Following high-dose infection, vaccinated animals showed stabilization of body weight from day 3 post-infection, in contrast to continued weight loss in the placebo group (Figure 3C). From day 6 post-infection, a trend towards weight gain was observed in all vaccinated animals (Figure 3D). After low-dose infection, a marked improvement was evident from day 5, with intensive weight gain in vaccinated animals. Unlike in the placebo group, body weight exceeded baseline values in this group by day 10 post-infection. These findings are consistent with other animal model studies, in which maximal weight loss occurs around days 6–7 post-infection, followed by spontaneous recovery and resolution of clinical signs by approximately day 14 [20,27,28].

Viral loads of SARS-CoV-2 in the lungs and nasal turbinates of infected Syrian hamsters were assessed over the course of experimental infection (Table 1 and Table 2).

In control animals (placebo group), SARS-CoV-2 viral titers on day 3 post-infection were comparable across both infection models, reaching 6.63 log_10_ TCID_50_/mL in the lungs and 6.13 log_10_ TCID_50_/mL in nasal turbinates. By days 6 and 10 post-infection, the infectious virus was below the limit of detection in all groups when assessed in Vero cells, which is consistent with previous studies showing a decline in viral load during the course of infection in hamsters [28,29,30].

Vaccinated animals showed reduced viral loads compared to the placebo group on day 3 post-infection. In the high-dose SARS-CoV-2 infection model, reductions in viral titres were 0.3 log_10_ in lung tissue and 0.8 log_10_ in nasal turbinates (Table 1). In the low-dose infection model, the reduction in lung viral load in vaccinated animals reached statistical significance compared to placebo controls, amounting to approximately 0.9 log_10_ in lung tissue (Table 2).

Importantly, no viral shedding was detected in the nasal turbinates of vaccinated hamsters, where the viral infectivity suppression exceeded 4.63 log_10_. This effective suppression of viral replication in the upper respiratory tract is likely attributable to the formation of local mucosal immunity following intranasal vaccination.

**Figure 4:**
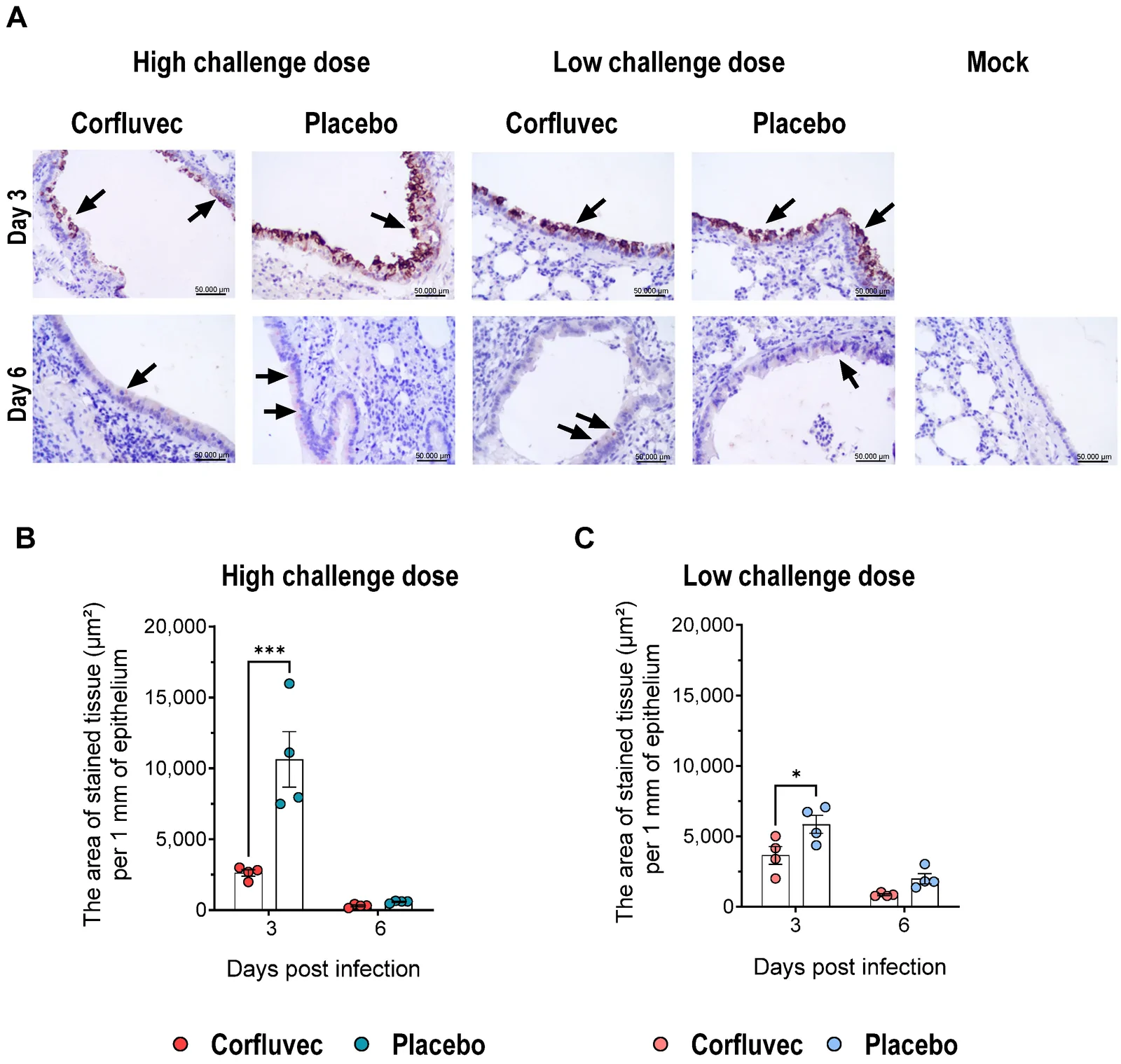
Immunohistochemical analysis of lung tissue from infected hamsters for SARS-CoV-2 S antigen. The target antigen is shown in brown (arrows); magnification ×400; scale bars = 50 µm. Variable staining intensity was observed, mainly in the airway epithelium (**A**). The area of immunopositive staining was quantified using ImageJ software. Data are presented as mean ± SEM (n = 4) for animals infected with high (**B**) and low (**C**) viral doses. Statistical significance was assessed using two-way ANOVA followed by Sidak’s multiple comparisons test. Differences were considered statistically significant at *p* < 0.05 (*: *p* < 0.05, ***: *p* < 0.001).

Expression of SARS-CoV-2 antigen in the airway epithelium of infected animals, including bronchi and, to a lesser extent, bronchioles and alveolar lung tissue, was confirmed by IHC staining for the SARS-CoV-2 S protein (Figure 4).

Morphometric analysis of IHC-stained lung sections showed that, by day 6 post-infection, viral antigen expression was significantly lower in all groups compared to the early stages of infection. This observation is consistent with previous reports indicating a decrease in viral load in infected hamsters over the course of infection [21,28].

Lower expression of SARS-CoV-2 antigen in vaccinated animals compared with the control group on day 3 post-infection (Figure 4A) indicated reduced virus replication in the bronchial and bronchiolar epithelium following *Corfluvec* administration. The most pronounced differences were observed in animals challenged with the high virus dose (*p* < 0.001; Figure 4B).

**Figure 5:**
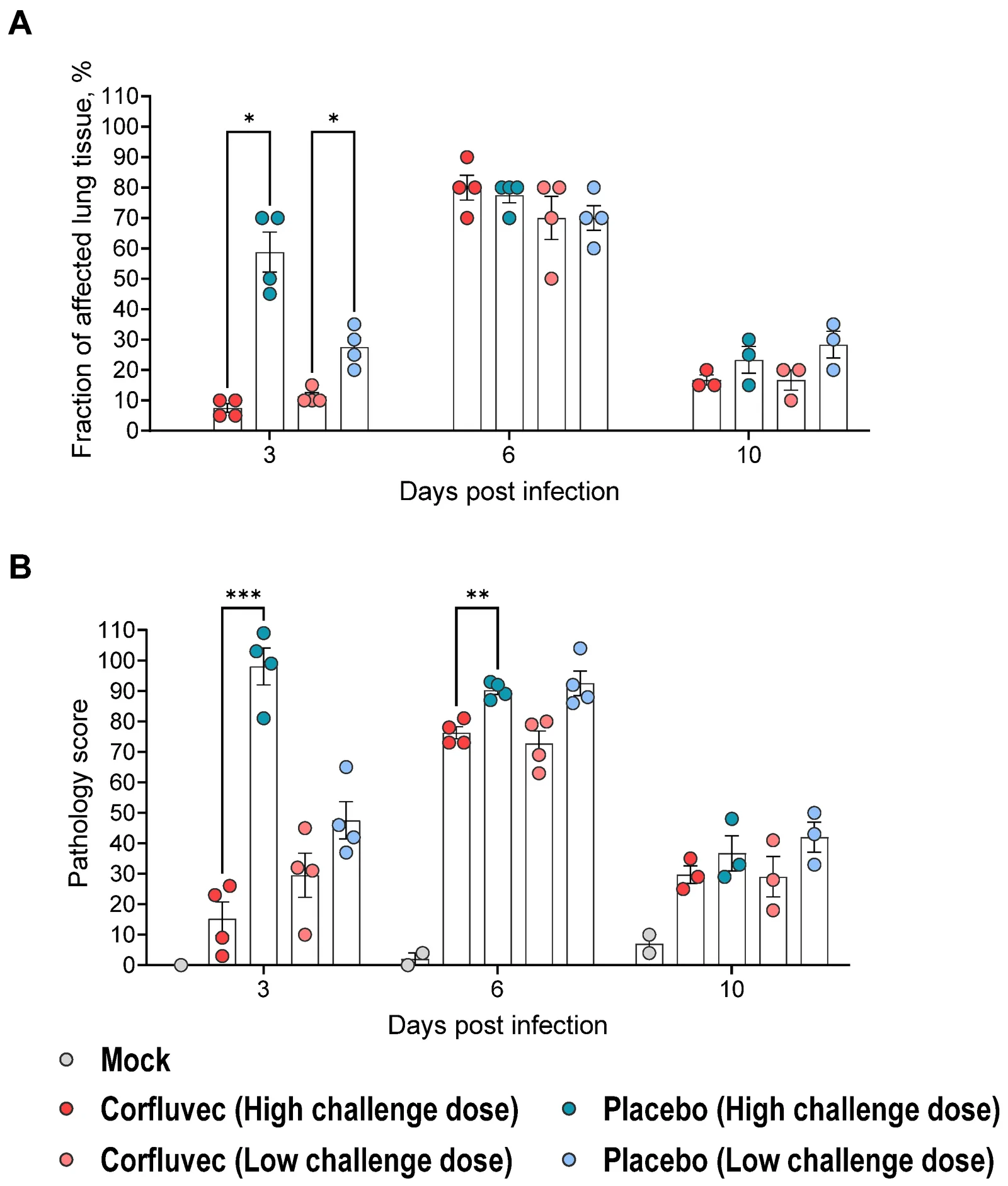
Semi-quantitative assessment of the severity of lung lesions in immunized hamsters following SARS-CoV-2 infection. The proportion of affected tissue (**A**) and overall pathology severity score (**B**) are shown. Data are presented as mean ± SEM, with *p*-values calculated using Sidak’s multiple comparisons test. Differences were considered statistically significant at *p* < 0.05 (*: *p* < 0.05, **: *p* < 0.01, ***: *p* < 0.001). Euthanasia of intact animals was not performed on day 3.

Pathomorphological changes varied in severity across groups and exhibited distinct features. A beneficial effect of the candidate vaccine on the course of SARS-CoV-2 infection was observed at both challenge doses. In the control group, on day 3 post-infection, the proportion of affected lung tissue ranged from 27.5% to 47.5%. By day 6, corresponding to the peak of infection, this increased to a median of 70–80%. By day 10, the proportion of affected lung tissue decreased to 15–30%, consistent with natural resolution of the pathological process. Compared with controls, vaccinated animals showed a smaller extent of pulmonary lesions on days 3 and 10 post-infection, with statistical significance observed on day 3 (Figure 5).

A composite histopathological score, which incorporated the severity of lung tissue damage, inflammation of structural components (bronchioles, vessels, stroma, and alveoli), tissue responses (epithelial hypertrophy and hyperplasia, epithelial necrosis, type II pneumocyte hypertrophy), and neutrophilic and lymphocytic infiltration, demonstrated a distinct decrease in overall pathological severity at all time points in vaccinated animals.

**Figure 6:**
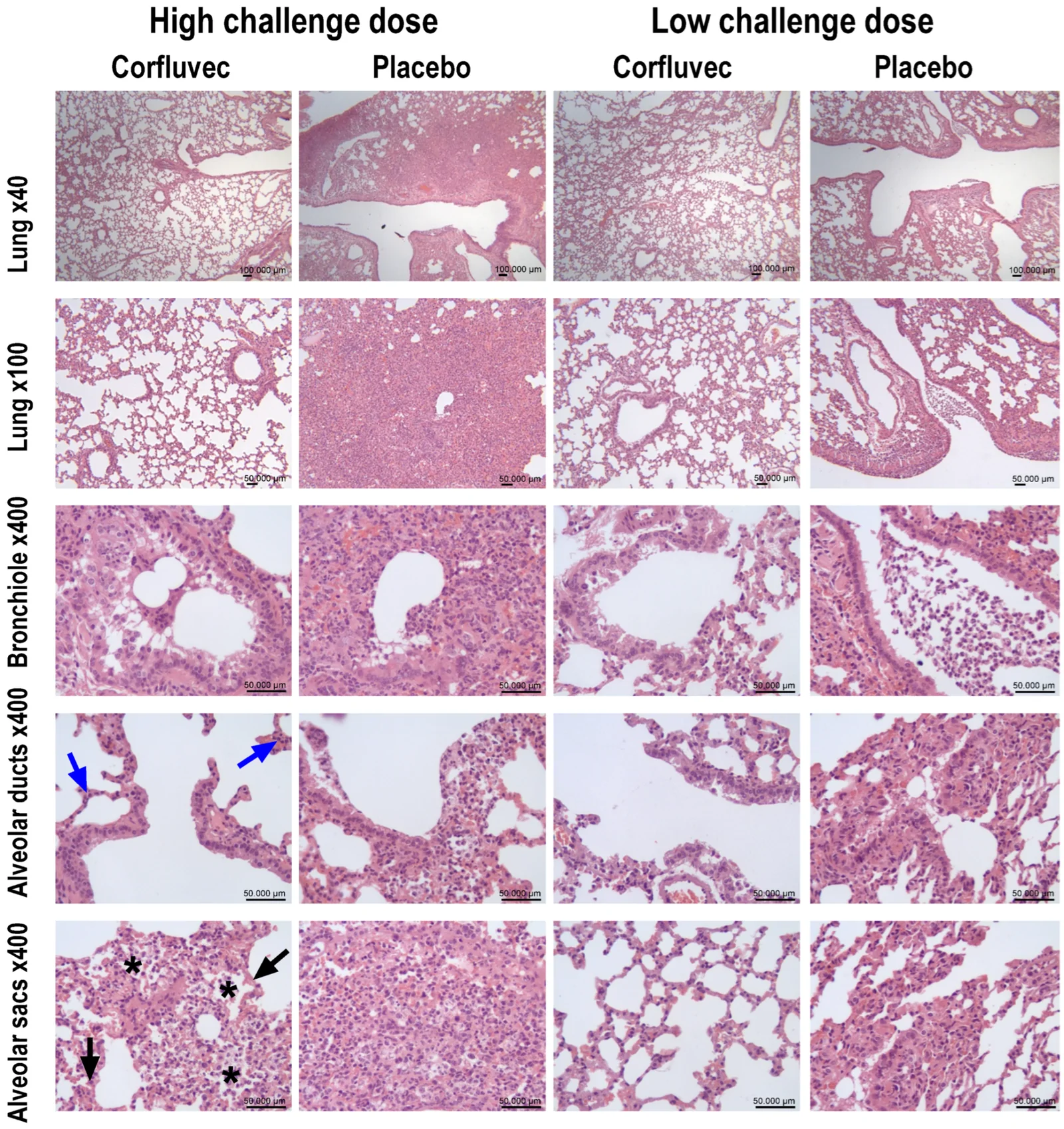
Microscopic structure of the lungs of immunized hamsters following SARS-CoV-2 infection. Representative microphotographs showing the most pronounced changes are presented. Day 3 post-infection, hematoxylin and eosin staining. Magnification ×40 (scale bars = 100 µm); magnification ×100 and ×400 (scale bars = 50 µm). The meaning of the arrows (black and blue) and asterisks is explained in the main text.

Figure 6 shows microphotographs of the most pronounced changes observed on day 3 post-infection. The corresponding microphotographs for days 6 and 10 are included in the Supplementary Materials (Figures S3 and S4).

In animals that received *Corfluvec* vaccine followed by challenge with a high dose of SARS-CoV-2, mild changes were observed: slight thickening of interalveolar septa (black arrows), focal lymphocytic infiltration (blue arrows), minimal alveolitis, and occasional macrophages and neutrophils in alveolar lumina (asterisks). In the high-dose placebo group, severe damage occurred—marked obstructive bronchopneumonia, hyaline membranes, bronchial/bronchiolar epithelial necrosis, diffuse inflammatory infiltration, vasculitis, and perivasculitis. Following low-dose challenge, vaccinated animals showed only mild bronchitis and bronchiolitis without alveolar involvement, whereas the low-dose placebo group exhibited moderately severe bronchitis, necrotic epithelial damage, focal alveolar septal infiltration, and occasional vasculitis. Notably, all vaccinated animals preserved lung aeration.

#### 3.1.2. *Corfluvec* Mitigates SARS-CoV-2-Induced Lung Vascular Damage

Severe COVID-19 is known to extend beyond the respiratory tract being closely associated with systemic manifestations, including microvascular injury and microthrombus formation driven by dysregulated inflammation and endothelial dysfunction [31,32].

**Figure 7:**
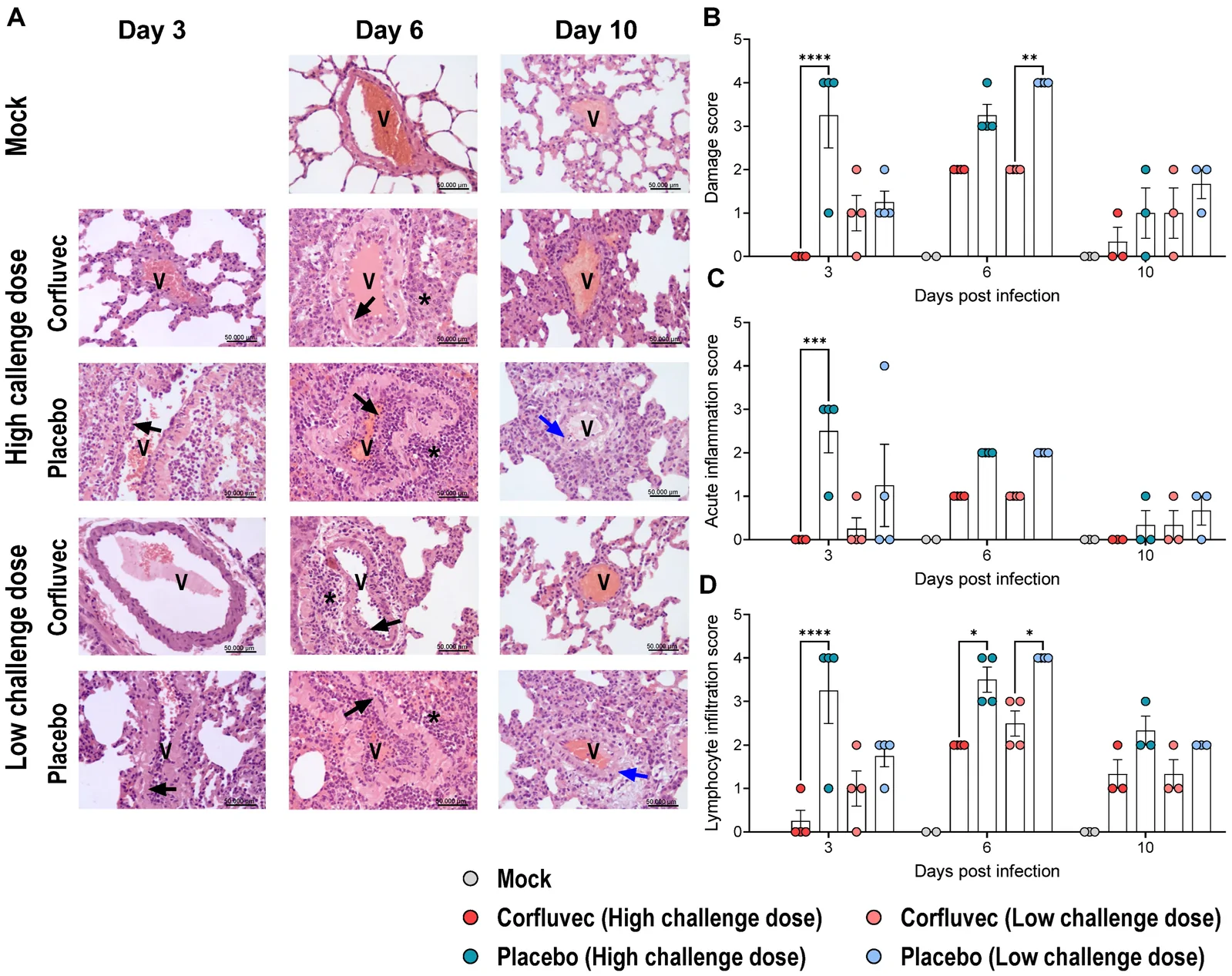
Histological evaluation of blood vessels in the lungs of infected hamsters. Microphotographs illustrating the most pronounced tissue damage (×400); scale bars = 50 µm. V indicates the lumen of the blood vessel; black arrows indicate endothelial lesions (loss of barrier integrity); asterisks indicate perivascular infiltration; blue arrows indicate perivascular edema (**A**). Tissue damage score (**B**). Acute inflammation score (**C**). Lymphocyte infiltration score (**D**). Data on panels (**B**–**D**) were considered statistically significant at *p* < 0.05 using two-way ANOVA followed by Tukey’s multiple comparisons test (*: *p* < 0.05, **: *p* < 0.01, ***: *p* < 0.001, ****: *p* < 0.001).

Histological analysis of lung vessels on days 3 and 6 post-infection revealed distinct, treatment-dependent differences (Figure 7). Vaccinated animals exhibited only mild vasculitis, characterized by minor endothelial hypertrophy and proliferation, with focal perivascular lymphocytic infiltration. In contrast, placebo controls showed significantly more severe vasculitis and perivasculitis, including extensive edema, diffuse infiltration of both large and small vessel walls, marked hypertrophy with disordered proliferation, focal dystrophic changes, and partial to complete loss of endothelial cells.

**Figure 8:**
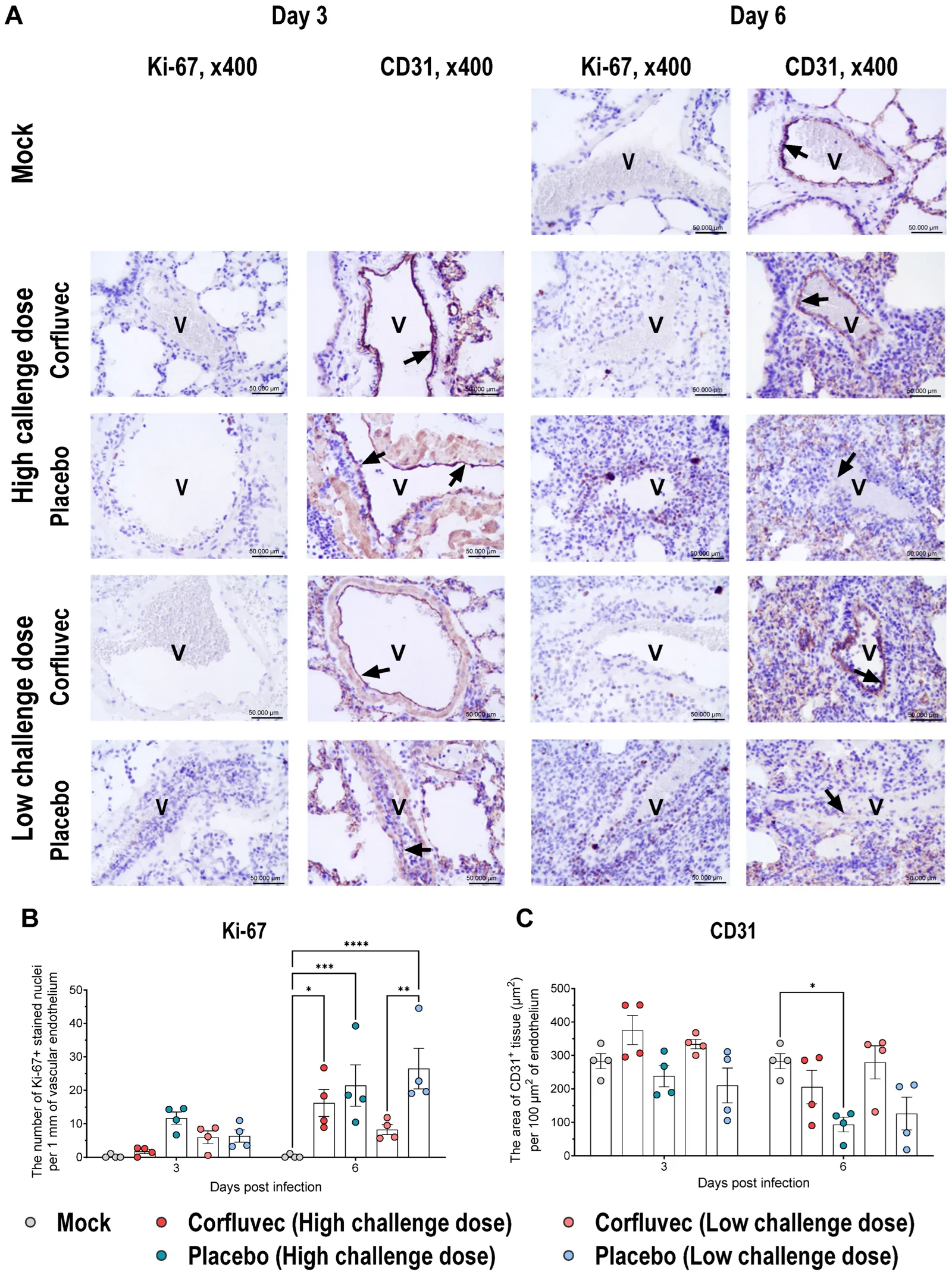
Immunohistochemical analysis of lung vascular tissue in immunized hamsters for Ki-67 and CD31 markers following SARS-CoV-2 infection. Representative microphotographs obtained on days 3 and 6 post-infection are shown; scale bars = 50 µm; V indicates the lumen of the blood vessel; black arrows indicate the inner surface of the vascular endothelium and the intensity of CD31 staining (**A**). Quantitative analysis of Ki-67 (**B**) and CD31 (**C**) staining. Cell counting/area measurements were performed in ImageJ software on individual tissue sections (mean ± SEM, n = 4). Data were considered statistically significant at *p* < 0.05 using two-way ANOVA followed by Sidak’s multiple comparisons test (*: *p* < 0.05, **: *p* < 0.01, ***: *p* < 0.001, ****: *p* < 0.001).

To investigate endothelium dysfunction, pulmonary vascular lesions were evaluated using IHC staining for Ki-67 and CD31 (Figure 8).

Ki-67 is considered a reliable marker of cellular proliferation [33], while CD31 (PECAM-1) serves as a primary indicator of endothelial cell integrity and density [34].

IHC analysis revealed significantly higher Ki-67 expression in the pulmonary vascular endothelium of placebo-treated hamsters compared with *Corfluvec*-vaccinated animals (Figure 8A,B). This pattern was observed on both day 3 and day 6 post-infection and was consistent with histological evaluation of blood vessels (Figure 7A) and semi-quantitative vasculitis scores (Figure 7B–D).

CD31 staining on day 3 showed a trend towards increased endothelial expression in vaccinated hamsters compared to intact controls, whereas placebo-treated animals had lower CD31 expression (Figure 8A,C). By day 6, a more significant decrease in CD31 expression was observed in placebo groups, reflecting progressive vascular inflammation associated with focal endothelial loss, dystrophic changes, and endothelial dysfunction. Notably, CD31 expression remained higher in vaccinated animals than in placebo controls at this time point. Together with the reduced severity of vascular inflammation, this suggests that *Corfluvec* can limit downstream endothelial damage and facilitate the preservation of lung vascular integrity in this model.

#### 3.1.3. Cross-Protective Efficacy of the *Corfluvec* Vaccine in a Lethal Murine Influenza Infection Model

To evaluate whether the vector-mediated immune response confers protection against influenza, a challenge study was conducted in outbred mice. This model is widely used for evaluating influenza vaccine efficacy, with established readouts including survival and weight loss following lethal challenge [35,36].

**Figure 9:**
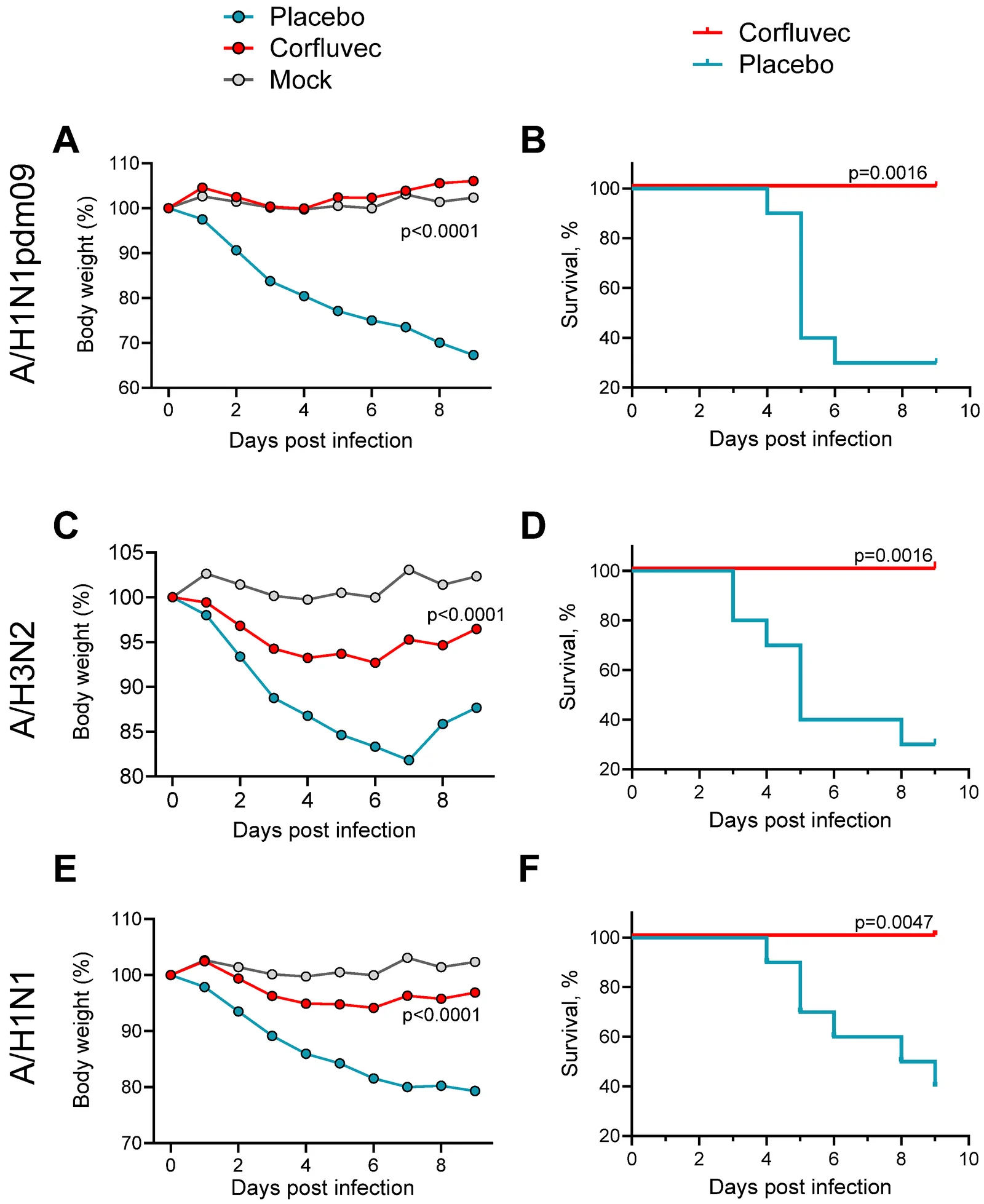
Protective efficacy of the candidate vaccine *Corfluvec* in a lethal murine influenza infection model. The degree of protection was assessed based on body weight dynamics and survival following challenge with mouse-adapted influenza strains: (**A**,**B**) A/California/07/2009 (H1N1pdm09), (**C**,**D**) A/Aichi/2/68 (H3N2), and (**E**,**F**) A/PR/8/1934 (H1N1). The graphs show *p*-values for comparisons between *Corfluvec* and placebo groups using ANOVA (Sidak’s test) for body weight (% change) and the Mantel–Cox test for survival. Data were considered statistically significant at *p* < 0.05.

Animals were immunized intranasally with two doses of *Corfluvec* (6.0 log_10_ TCID_50_/30 µL per dose), administered 21 days apart. Placebo and mock groups received placebo administered in the same regimen. Twenty-eight days after the second *Corfluvec* dose, mice were challenged i.n. with wild-type influenza viruses (10 MLD_50_/30 μL per animal) using mouse-adapted strains: homologous—A/California/07/2009 (H1N1pdm09), antigenically drifted—A/Aichi/2/68 (H3N2), and heterologous—A/PR/8/1934 (H1N1) (Figure 9).

*Corfluvec* immunization provided protection against lethality under both homologous and heterologous challenge conditions (Figure 9B,D,F). In contrast, mortality in the placebo-treated groups reached 70% following infection with A/California/07/2009 (H1N1pdm09) or A/Aichi/2/68 (H3N2), and 60% after A/PR/8/1934 (H1N1) infection. Placebo-treated mice lost 20–40% of their body weight post-infection, whereas *Corfluvec*-vaccinated mice exhibited no weight loss following homologous A/H1N1pdm09 challenge (Figure 9A); heterologous challenges with A/H3N2 or A/H1N1 resulted in only minimal weight loss (5–7%; Figure 9B,C). These findings demonstrate that *Corfluvec* confers protection against lethal infection with both homologous and antigenically distinct heterologous influenza viruses.

### 3.2. Evaluation of Corfluvec Immunogenicity in Non-Human Primates

Since *Corfluvec* is intended for intranasal administration, it was deemed essential to evaluate its immunogenicity in larger animals than rodents, permitting the use of a human-compatible delivery device. In this regard, NHP (non-human primates) represent the most relevant model. Although the NHP model of COVID-19 does not fully recapitulate protective efficacy due to differences in disease severity and clinical outcomes, it remains highly informative for safety and immunogenicity assessments and is substantially more relevant than rodents for mucosal vaccine studies [37].

**Figure 10:**
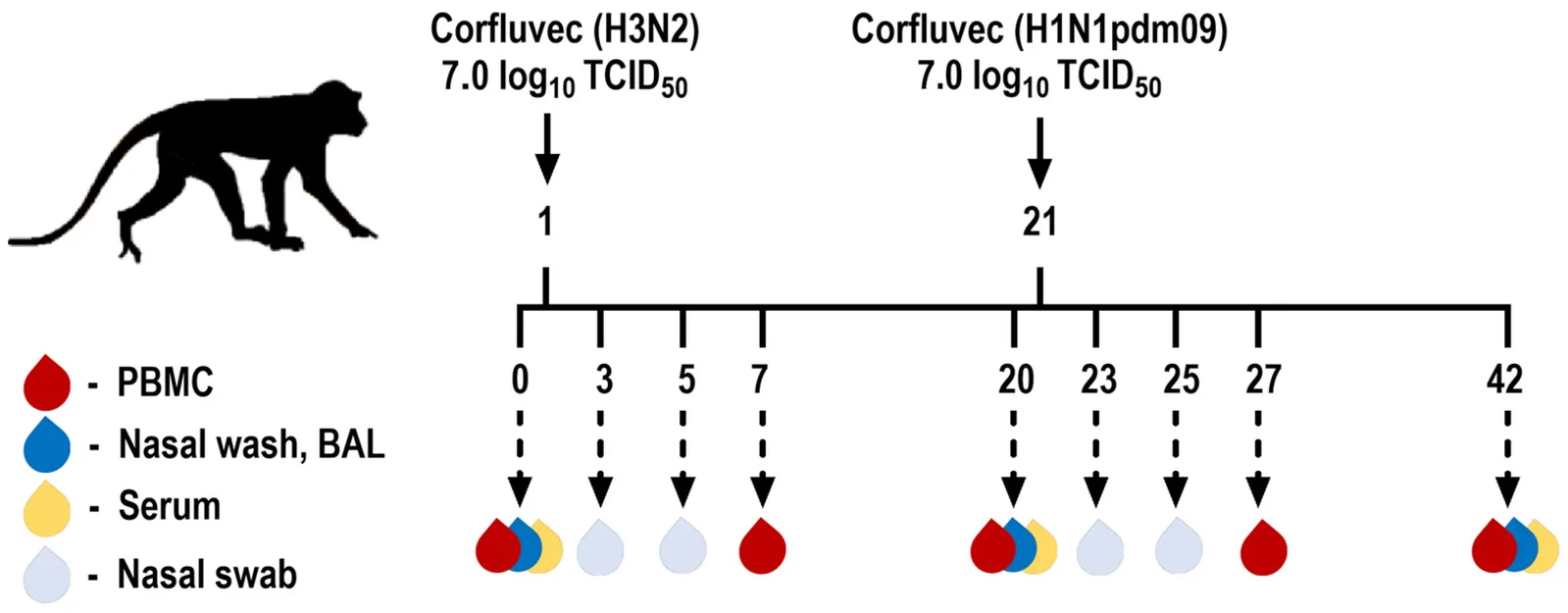
*Corfluvec* safety and immunogenicity in NHP: study design and sample collection. Four male *Macaca fascicularis* were immunized i.n. with each component of the *Corfluvec* vaccine (0.5 mL, 7.5 log_10_ TCID_50_/animal), administered at three-week intervals. Two control animals (1 male and 1 female *Macaca fascicularis*) received placebo (stabilizing buffer) following the same regimen. Blood collection, nasal swabs, and bronchoalveolar lavage (BAL) fluid collection were performed at indicated time-points to evaluate local and systemic immune responses to vaccination. Body weight and temperature were measured before the first immunization and on days 2, 4, 8, 21, and 42. To evaluate the vaccine virus shedding, nasal swabs were collected from macaques on days 3 and 5 after each immunization and analyzed by RT-PCR.

According to the study design (Figure 10), four adult male macaques aged 2.5–6 years were immunized i.n. with each component of the *Corfluvec* vaccine (0.5 mL, 7.5 log_10_ TCID_50_/animal), administered via a human-compatible syringe-based nasal sprayer at three-week intervals. Two additional animals, one male and one female, received placebo (stabilizing buffer) following the same regimen.

No adverse clinical signs were observed throughout the study. Body weight temperature and clinical biochemistry parametrs remained within physiological ranges [38,39], with no vaccine-related deviations (Supplementary Tables S2–S5). Importantly, vaccine virus replication was strictly limited to the administration site: RT-PCR detected influenza viral RNA in nasal swabs on day 3 after each immunization, but shedding was undetectable by day 5, confirming the restricted replicative capacity of the vaccine strains in the primate respiratory tract (Table 3).

**Figure 11:**
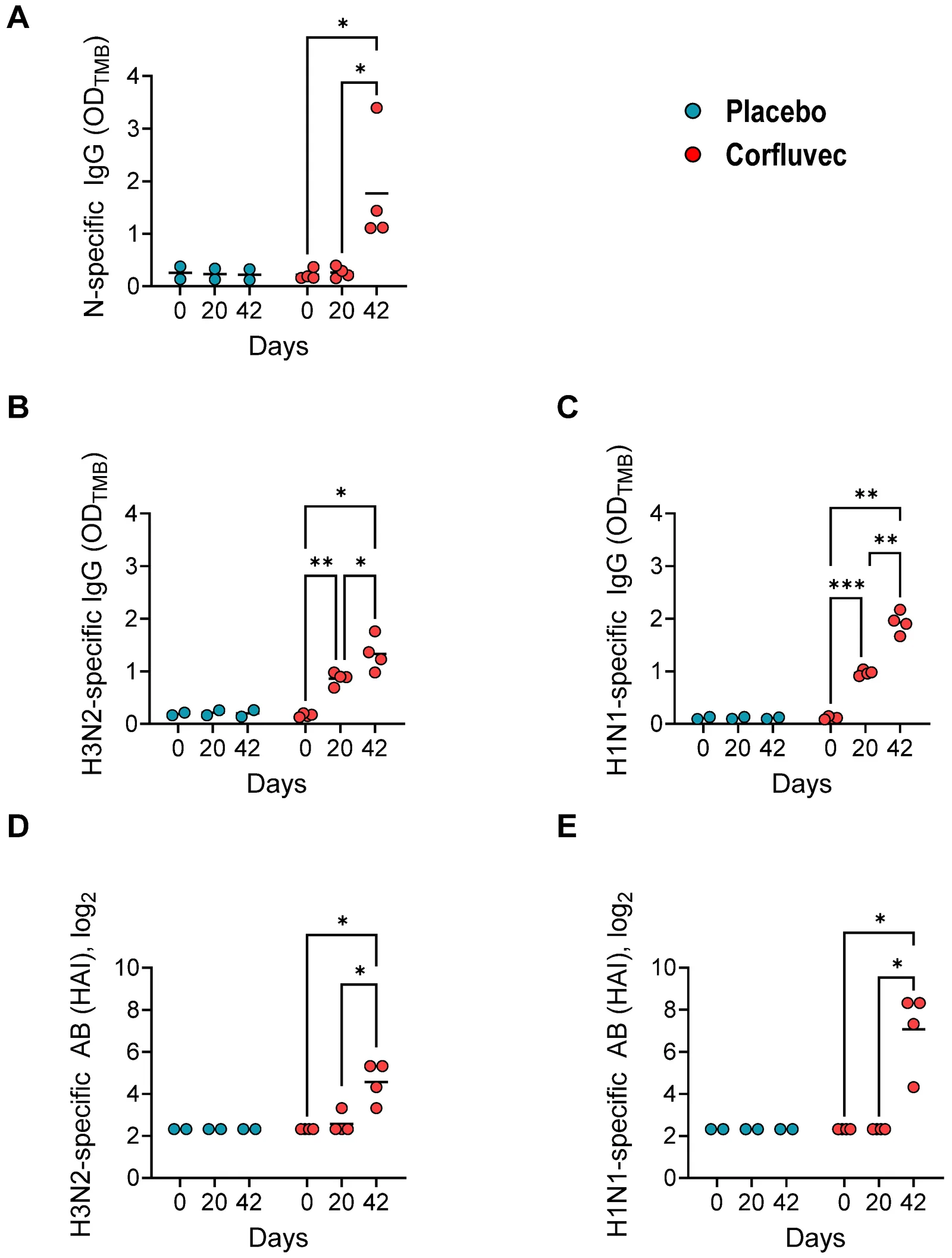
Virus-specific serum IgG levels in cynomolgus macaques before and after *Corfluvec* immunization: anti-SARS-CoV-2 N protein (**A**), influenza A/H3N2 (**B**), and A/H1N1pdm09 (**C**). HAI titers for H3N2 (**D**) and H1N1pdm09 (**E**)-specific antibodies are also shown. Data are presented as log_2_ titers. Statistical significance was defined as *p* < 0.05 using two-way RM ANOVA followed by Tukey’s multiple comparisons test (*: *p* < 0.05, **: *p* < 0.01, ***: *p* < 0.001).

The post-vaccination humoral immune response was assessed by measuring antigen-specific systemic IgG antibodies in serum and local IgA antibodies in nasal washes and BAL fluid using enzyme-linked immunosorbent assay (ELISA). Following two vaccine doses, all four macaques showed a statistically significant increase in IgG antibody levels against the SARS-CoV-2 N protein (Figure 11A), whereas no antibody response was detected in placebo-treated controls. Systemic IgG levels specific to influenza A/H1N1 and A/H3N2 increased significantly after a single immunization and rose further after the second dose (Figure 11B,C). HAI titers against both vaccine antigens were detectable after the second immunization (Figure 11D,E).

**Figure 12:**
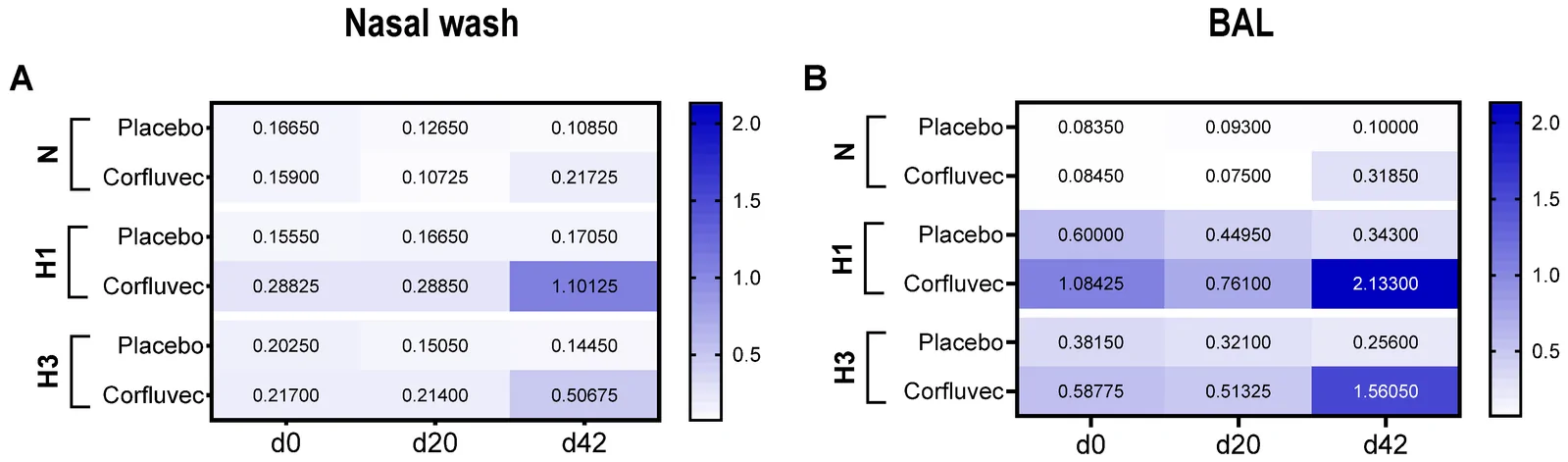
Heat map of virus-specific IgA antibody levels (OD_TMB_) in the nasal washes (**A**) and BAL fluid (**B**) of cynomolgus macaques before and after *Corfluvec* immunization: anti-SARS-CoV-2 N protein (N), influenza A/H1N1 (H1), and A/H3N2 (H3).

N protein-specific local IgA antibodies were detected in BAL fluid and nasal secretions in two of four macaques immunized twice with the vaccine. Influenza-specific local antibodies were observed in three of four vaccinated animals (Figure 12A,B).

These findings may reflect animal heterogeneity and are consistent with published data showing local antibody responses in only a subset of rhesus macaques, even after confirmed SARS-CoV-2 infection [38]. However, in a prime-boost immunization study in which rhesus macaques received an adjuvanted subunit vaccine first intramuscularly and then intranasally, local IgA and IgG antibodies were detected in all animals. Intranasal boosting was more effective at inducing specific dimeric IgA antibodies in BAL fluid and prevented SARS-CoV-2 replication in the nasal cavity, unlike a three-dose intramuscular regimen of the candidate vaccine [40].

IFNγ production in response to PepTivator N stimulation was assessed by ELISPOT in cryopreserved PBMCs collected at baseline (day 0) and on days 7, 20, 27, and 42 after the first and second vaccinations. IFNγ response was considered positive if 10 or more spots were detected in response to antigen-specific stimulation.

**Figure 13:**
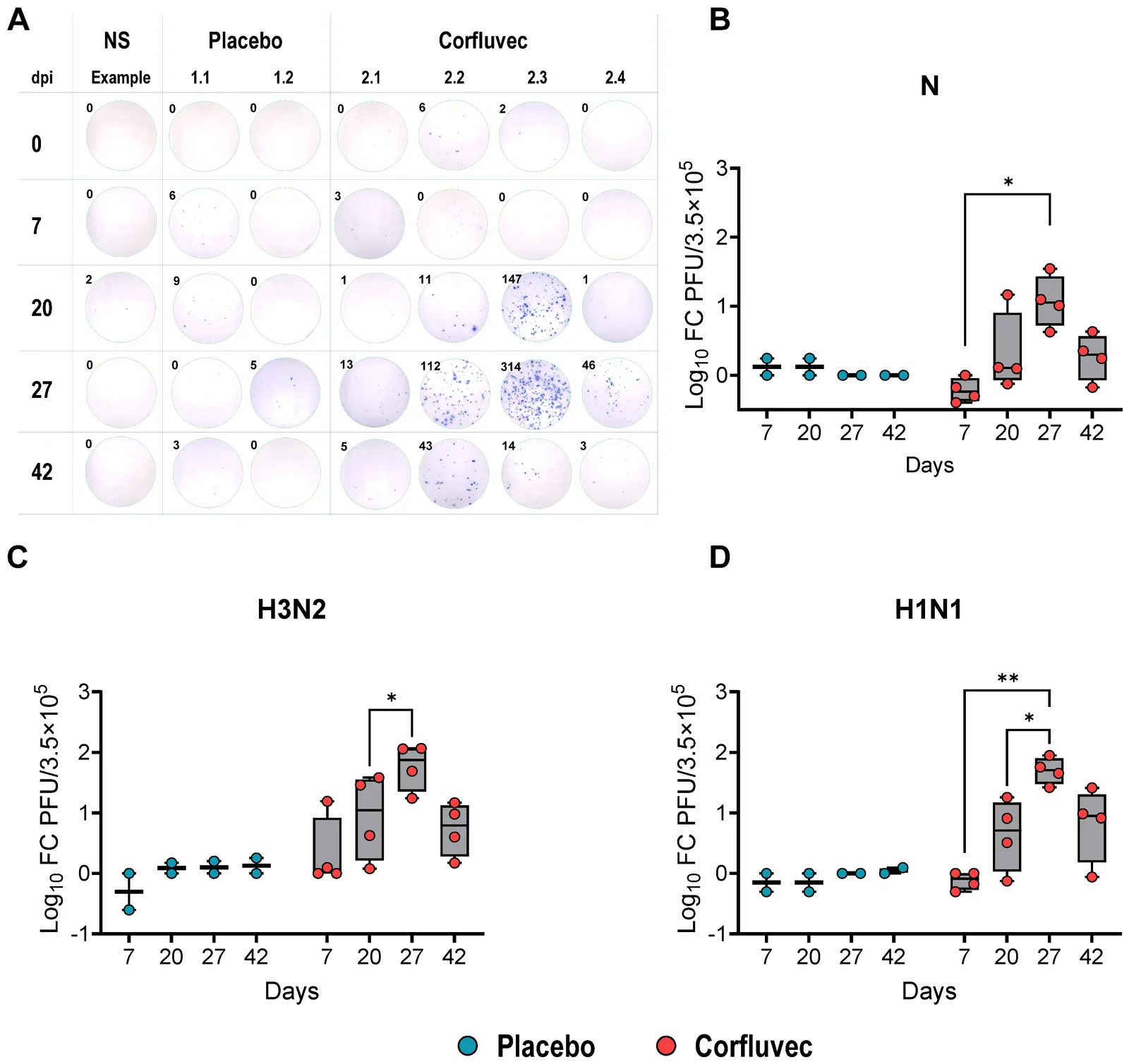
T-cell response to *Corfluvec* vaccination in cynomolgus macaques (*Macaca fascicularis*). PBMCs (3.5 × 10^5^/100 µL/well) were stimulated with PepTivator^®^ SARS-CoV-2 Prot_N (5 µg/mL) and H3N2 and H1N1pdm09 monovalent split vaccine preparations (5 µg/mL) for 24 h. IFNγ release was assessed by ELISPOT assay. N-specific response: raw ELISPOT data (**A**); individual normalized fold-change values (log_10_ FC) in spot counts following stimulation with PepTivator^®^ SARS-CoV-2 Prot_N (**B**), influenza H3N2 (**C**) and influenza H1N1pdm09 (**D**) relative to baseline. Data on panels B-D are presented as gray box-and-whisker plots showing min–max ranges with individual values overlaid Statistical significance was defined as *p* < 0.05 using two-way RM ANOVA followed by Tukey’s multiple comparisons test (*: *p* < 0.05, **: *p* < 0.01).

After the first vaccination, a specific IFNγ response was observed in two of four vaccinated animals (day 20, before the second dose; Figure 13A). Following the second dose, IFNγ-producing cells were detected in all vaccinated animals, peaking seven days after revaccination (day 27; Figure 13B). At this time point, the fold increase in antigen-specific IFNγ-producing cells relative to baseline (day 0) ranged from 9 to 160 across individual animals.

T-cell responses to vaccine vectors were detected in all immunised animals and demonstrated the same dynamics as N-specific responses (Figure 13C,D).

The observed decline in IFNγ-producing cells by day 42 is likely due to the redistribution of memory T cells from systemic circulation to peripheral tissues, as reported in other studies [40].

These results indicate that a two-dose intranasal vaccination regimen with the candidate vaccine *Corfluvec* (H3N2 → H1N1) in Macaca fascicularis is well tolerated and induces both systemic and local immune responses against SARS-CoV-2 and influenza antigens. This is supported by the detection of antigen-specific serum IgG and secretory IgA (sIgA) in vaccinated animals. In addition to humoral responses, vaccination also elicited SARS-CoV-2-specific T-cell immunity. Previous studies on intranasal vaccines in primate models of experimental SARS-CoV-2 infection have demonstrated their ability to induce sIgA production at the site of infection [39]. Compared to parenteral vaccines, mucosal vaccines provide stronger protection and are associated with reduced viral shedding in the upper respiratory tract [37,40].

## 4. Discussion

The concurrent circulation of SARS-CoV-2 and influenza viruses represents a growing public health concern. Simultaneous or combined vaccination strategies have emerged as effective approaches to streamline immunization schedules, broaden cross-protective coverage, and alleviate the burden on global healthcare systems [15].

From this perspective, influenza virus vectors expressing SARS-CoV-2 antigen(s) offer a pragmatic and scalable solution. Immunization with live vectored vaccines elicits robust multifaceted immune responses against both the influenza backbone and the encoded heterologous antigen [41]. Influenza-vectored vaccines can be administered easily and non-invasively via nasal sprays or nebulization. This needle-free approach is associated with fewer systemic adverse reactions and significantly improves vaccine acceptance among diverse social groups [42]. Moreover, intranasal vaccination enhances protection against respiratory pathogens by stimulating mucosal immune responses at the primary site of viral infection, thereby facilitating a balanced and effective immune response. Importantly, live vectored vaccines promote the early production of cytokines and chemokines as part of the defense inflammatory response and induce trained innate immunity [21,22,23] which provides a self-adjuvanting effect. This is clinically meaningful, as mucosal adjuvants have historically faced safety concerns, including Bell’s palsy associated with an adjuvanted intranasal influenza vaccine withdrawn from the Swiss market [43] and neurological issues reported with detoxified derivatives [44]. These safety issues have been a major barrier to the clinical translation of adjuvanted mucosal vaccines. The absence of an exogenous adjuvant therefore not only simplifies the formulation but also enhances its safety profile, particularly for a vaccine intended for use in healthy populations.

This conceptual framework has driven the development of several influenza-vectored vaccine candidates, including dNS1-RBD (Pneucolin) [45,46], ΔNA(RBD)-Flu [47], LAIV/CoV-2 [48], and FluCoV-N [49], each expressing SARS-CoV-2 antigens from different open reading frames (ORFs) within the influenza genome. In preclinical animal studies, intranasal administration of these constructs has demonstrated a favorable safety and immunogenicity profile, providing dual protection against both SARS-CoV-2 and influenza. Notably, dNS1-RBD (Pneucolin) was the first intranasal mucosal vaccine to receive Emergency Use Authorization (EUA) in China and has successfully progressed through clinical trials [50,51], proving to be well-tolerated even in pediatric cohorts [52]. In a Syrian hamster model, dNS1-RBD conferred broad protection against antigenically distant SARS-CoV-2 XBB variants [46]. However, its efficacy against Omicron infection in a Phase 3 clinical trial was modest (28.2%, 95% CI 3.4–46.6) [51]. Given the unprecedented evolutionary rate of the spike RBD domain—characterized by frequent mutations within key B- and T-cell epitopes under selective immune pressure [53,54], the long-term antigenic relevance of RBD-targeted vaccines may be constrained. In contrast, the highly conserved nucleocapsid (N) protein represents a more stable vaccine target [7,55,56].

The *Corfluvec* vaccine is based on an influenza A vector with a partially truncated NS1 gene encoding the SARS-CoV-2 N protein. This NS1 modification enables the use of traditional, well-established and cost-effective egg-based manufacturing technology while maintaining a high degree of attenuation, robust immunogenicity, and protective efficacy. Previously, we showed that a two-dose intranasal immunization regimen with prototype vaccine strains protected naïve mice against SARS-CoV-2 challenge despite the absence of detectable serum neutralising antibodies. The observed protection was associated with local virus-specific immunity and the suppression of the inflammatory response upon infection [14].

In the present study, first we have shown using Syrian hamster model that even single-dose immunization in naïve animals is protective. However, the main focus was on the study of antigenically updated H3N2 and H1N1 vector strains which were used in a sequential heterologous prime-boost regimen.

In the Syrian hamster model, the two-component intranasal vaccination with *Corfluvec* improved disease outcomes following SARS-CoV-2 challenge (B.1.1 lineage), resulting in a significantly reduced viral load in the lungs compared to the placebo control group. Notably, following the low-dose SARS-CoV-2 challenge, viral shedding from the nasal turbinates was rendered undetectable in vaccinated animals, consistent with the induction of local mucosal immunity as a result of intranasal vaccination. Furthermore, vaccination markedly reduced the severity of lung inflammation and tissue damage at both challenge doses without eliciting any signs of vaccine-associated enhanced respiratory disease (VAERD).

Since SARS-CoV-2-induced vascular endothelium damage acts as a primary driver of severe COVID-19 pathology [24,25], our evaluation focused on the vaccine’s capacity to mitigate this specific tissue damage. The vascular endothelium is a primary target of the systemic inflammatory response triggered by SARS-CoV-2. In severe clinical cases, viral infection and the resulting cytokine storm may lead to endotheliitis characterized by profound endothelial dysfunction, loss of barrier integrity, and microthrombus formation [57,58]. In this study, vaccinated hamsters exhibited a significant preservation of vascular integrity during SARS-CoV-2 infection. Importantly, the preservation of vascular and endothelial integrity in *Corfluvec*-vaccinated hamsters during SARS-CoV-2 infection was accompanied by sustained high CD31 expression, suppressed Ki-67 proliferative activity, and less severe lung tissue damage. Since the SARS-CoV-2 spike (S) protein is documented to directly induce endothelial dysfunction via ACE2 downregulation and mitochondrial fragmentation [59], targeting alternative viral antigens potentially represents a viable vaccination strategy to mitigate downstream vascular complications.

CD31 (PECAM-1) is a constitutive marker of endothelial integrity, playing a critical role in maintaining vascular barrier function and preventing vascular leakage [34,60]. Its sustained expression in *Corfluvec*-vaccinated hamsters is therefore indicative of preserved endothelial integrity, which is essential for preventing edema and vascular leakage—hallmarks of severe SARS-CoV-2-induced pathology [31,32]. Ki-67, a marker of cellular proliferation, is not expressed in quiescent endothelium; its upregulation in the placebo group reflects a proliferative repair response driven by endothelial injury and inflammatory stress [33,61]. This elevation is consistent with vascular remodeling triggered by SARS-CoV-2 infection and the associated cytokine storm [62]. In contrast, the suppression of Ki-67 expression in vaccinated animals suggests that the vaccine mitigated endothelial damage, thereby reducing the need for compensatory proliferative repair and preserving normal vascular homeostasis. Together, these findings indicate that *Corfluvec* not only limits viral replication but also protects against the vascular complications of SARS-CoV-2 infection, likely through the induction of local immune responses that reduce endothelial activation and inflammatory damage.

*Corfluvec* was also shown to confer protection against both homologous and heterologous lethal influenza infection in mice. This cross-protection is presumably mediated by T-cell response directed against influenza conserved proteins. The ability of the prototype *Corfluvec* vaccine vector (FluVec-N) to stimulate influenza NP-specific T-cell response in a mouse model was shown previously [14]. Our data align with previous reports detailing the cross-protective properties of NS1-truncated influenza viruses [22,63] and confirm that the insertion of the SARS-CoV-2 N-protein antigen does not compromise this effect.

In *Macaca fascicularis* two-dose intranasal regimen of *Corfluvec* administration via human-compatible delivery device was found to be immunogenic. Systemic antibody and T-cell responses to the influenza backbone were detected after the first vaccine dose and were boosted following the second administration. The observed unexpected increase in H3N2 HAI titers following the booster H1N1 immunization may reflect a suboptimal priming by the first *Corfluvec* dose in naïve animals. In the absence of detectable HAI antibodies, vaccine uptake was confirmed by positive RT-PCR. The second vaccine dose apparently provided a heterologous CD4^+^ T-cell mediated boost. Two immunizations were required to elicit SARS-CoV-2 N-specific systemic IgG and local secretory IgA antibodies in BAL fluids and nasal secretions as well as T-cell response in peripheral blood in immunologically naïve animals. The safety profile of *Corfluvec* was preliminarily assessed. The vaccine was well tolerated, with no adverse effects on clinical condition, body weight, or temperature, and vaccine virus replication was strictly restricted to the nasal epithelium.

Despite these promising insights, several limitations must be acknowledged. A key limitation is the absence of efficacy data for antigenically divergent, recently emerged SARS-CoV-2 variants. However, the C-terminal fragment of the N protein targeted by *Corfluvec* (amino acids 211–369) is highly conserved across Omicron subvariants [64], providing a rational basis for T-cell-mediated cross-reactivity. This is further corroborated by in silico analyses showing that CD8^+^ T-cell epitopes in immunodominant regions of the N protein are partially retained across Omicron variants [65], raising the possibility that cross-protective responses may be elicited even in the context of an early B.1.1 challenge. The B.1.1 strain was selected for this study because it was the dominant circulating lineage at the time of study initiation, carrying the D614G mutation. Nevertheless, direct evidence of vaccine efficacy against contemporary strains remains to be established in future studies.

Another limitation concerns on the small cohort size in NHP study (n = 4 vaccine, n = 2 placebo) constrains the interpretability of the mucosal IgA and T-cell responses, particularly given the inherent biological variability in outbred non-human primates. These results should therefore be viewed as exploratory, rather than as definitive evidence of efficacy or correlates of protection. Nevertheless, the data provide valuable proof-of-concept evidence that intranasal immunization with a human-compatible delivery device—a nasal atomizer approved for use in humans—can induce both local mucosal (sIgA) and systemic T-cell responses in a large animal model closely related to humans.

Most of the current population is seropositive, bearing systemic anti-Spike antibodies from prior vaccination or infection. However, this systemic antibody-mediated protection barrier has proven insufficient to halt breakthrough transmission or fully prevent lung and vascular damage caused by emerging variants. Our results demonstrate that immunization with *Corfluvec* reduced viral load (including complete suppression of nasal shedding upon low-dose challenge) and decreased lung pathology, leading to the preservation of pulmonary endothelial and vascular integrity. By targeting the highly conserved N antigen via the mucosal route, administration of *Corfluvec* could enhance and broaden the pre-existing immunity. Furthermore, given the continued co-circulation and evolution of influenza and SARS-CoV-2, and the need for regular revaccination, developing a single-dose immunization regimen may be warranted.

## 5. Conclusions

Our findings suggest that *Corfluvec* is a promising bivalent vaccine candidate that provides dual protection against influenza and SARS-CoV-2. The preclinical data have enabled the initiation of a randomized, double-blind, placebo-controlled Phase 1/2 clinical trial of *Corfluvec* (“Evaluation of the *Corfluvec* Vaccine for the Prevention of COVID-19 in Healthy Volunteers”; ClinicalTrials.gov ID: NCT05696067), assessing the intranasal vectored vaccine for the prevention of COVID-19 in healthy volunteers aged 18 to 60 years. The study enrolled 200 participants, with a follow-up period of approximately 6.5 months. The results are currently being prepared for publication.

## Figures and Tables

**Table 1:** Virus titers in the respiratory tract of immunized and control hamsters following high-dose SARS-CoV-2 infection.

Group	Day After Challenge					
	Day 3		Day 6		Day 10	
	Lung (n = 4)	Nose (n = 4)	Lung (n = 4)	Nose (n = 4)	Lung (n = 3)	Nose (n = 3)
Vaccine	6.28 ± 0.38	5.20 ± 1.71	<2.00	<2.00	<2.00	<2.00
Placebo	6.58 ± 0.34	6.03 ± 0.38	<2.00	<2.00	<2.00	<2.00
Virus infectivity suppression index, Δlog_10_	0.30	0.83	nd	nd	nd	nd

Note: The values of virus infectivity in log_10_ TCID_50_/mL of tissue suspension are presented, mean ± SD; <2.00 is below the limit of detection; nd—not determined.

**Table 2:** Virus titers in the respiratory tract of immunized and control hamsters following low-dose SARS-CoV-2 infection.

Group	Day After Challenge					
	Day 3		Day 6		Day 10	
	Lung (n = 4)	Nose (n = 4)	Lung (n = 4)	Nose (n = 4)	Lung (n = 3)	Nose (n = 3)
Vaccine	5.75 ± 0.84	<2.00	<2.00	<2.00	<2.00	<2.00
Placebo	6.63 ± 0.47	6.13 ± 0.15	<2.00	<2.00	<2.00	<2.00
Virus infectivity suppression index, Δlog_10_	0.88 ^†^	>4.63 *	nd	nd	nd	nd

Note: The values of virus infectivity in log_10_ TCID_50_/mL of tissue suspension, mean ± SD, are presented; <2.00, below the limit of detection; nd—not determined; †—statistically significant difference with the placebo group, *p* = 0.05; *—statistically significant difference with the placebo group at *p* < 0.0001.

**Table 3:** Results of RT-PCR analysis of nasopharyngeal swab samples from cynomolgus macaques following the first (days 3 and 5) and second (days 23 and 25) vaccinations.

Group	Animal No.	Study Days			
		3	5	23	25
		Nasopharyngeal Swab RT-PCR Results (Ct)			
Placebo	1.1	Neg.	Neg.	Neg.	Neg.
	1.2	Neg.	Neg.	Neg.	Neg.
Corfluvec	2.1	Pos. (23.39)	Neg.	Pos. (19.47)	Neg.
	2.2	Pos. (22.11)	Neg.	Pos. (21.70)	Neg.
	2.3	Pos. (18.57)	Neg.	Pos. (20.20)	Neg.
	2.4	Pos. (19.96)	Neg.	Pos. (20.32)	Neg.

Neg.: negative; Pos.: positive (Ct value).

## Data Availability

The data presented in this study are available on reasonable request from the corresponding author. The data are not publicly available due to the institution’s policy.

## Supplementary Materials

The following supporting information can be downloaded at: https://www.mdpi.com/article/10.3390/vaccines14080684/s1, Table S1—Overview of animal studies, immunization regimens, and challenge conditions; Figure S1. Workflow for the identification, imaging, and quantification of immunopositive areas; Figure S2. Semi-quantitative assessment of the severity of lung lesions in FluVec_N (H1N1pdm) immunized hamsters following SARS-CoV-2 infection. Figure S3. Microscopic structure of the lungs of immunized hamsters following SARS-CoV-2 infection day 6. Figure S4. Microscopic structure of the lungs of immunized hamsters following SARS-CoV-2 infection day 10. Table S2—Body weight of *cynomolgus macaques*, individual readings; Table S3—Body temperature of *cynomolgus macaques*, individual readings measured at three sites (forehead, nose, rectal); Table S4—Hematological parameters of *cynomolgus macaques*, individual values. Table S5—Biochemical parameters of *cynomolgus macaques*, individual values.
