# Supplementary material for: Novel Intranasal Influenza-Vectored Vaccine Corfluvec Provides Protection Against Influenza and COVID-19, Mitigating SARS-CoV-2-Induced Lung Vascular Damage
Source: Vaccines (Basel). 2026 Aug 8;14(8):684. doi: 10.3390/vaccines14080684 (PMC13517564; doi:10.3390/vaccines14080684)
Supplement: Supplementary file 1 [file vaccines-14-00684-s001.zip › vaccines-4442514-supplementary.pdf]

vaccines

MDPI

Supplementary

Table S1 – Overview of animal studies, immunization regimens, and challenge conditions.

Study	Animal model	n per group	Vaccine strain(s)	Doses	Challenge strain(s)
SARS-CoV-2 efficacy (preliminary)	Syrian hamster	6	FluVec-N (H1N1pdm09) or Flu_NS124 - an empty vector (H1N1pdm09)	1	SARS-CoV-2 isolate 11308A, clade GR, lineage B.1.1 (5.0 log_{10} $TCID_{50}$/0.1 mL/animal)
SARS-CoV-2 efficacy (main)	Syrian hamster	11 (vaccine/placebo), 5 (intact)	*Corfluvec* (FluVec-N (H3N2) → FluVec-N (H1N1pdm09))	2	SARS-CoV-2 isolate 11308A, clade GR, lineage B.1.1 (4.5 or 3.0 log_{10} $TCID_{50}$/0.026 mL/animal)
Influenza efficacy	Mouse	10	*Corfluvec* (FluVec-N (H3N2) → FluVec-N (H1N1pdm09))	2	A/California/07/2009 (H1N1pdm09), A/Aichi/2/68 (H3N2), A/PR/8/1934 (H1N1)
Immunogenicity	NHP (*Cynomolgus macaque*)	4 (vaccine), 2 (placebo)	*Corfluvec* (FluVec-N (H3N2) → FluVec-N (H1N1pdm09))	2	no challenge

vaccines

MDPI

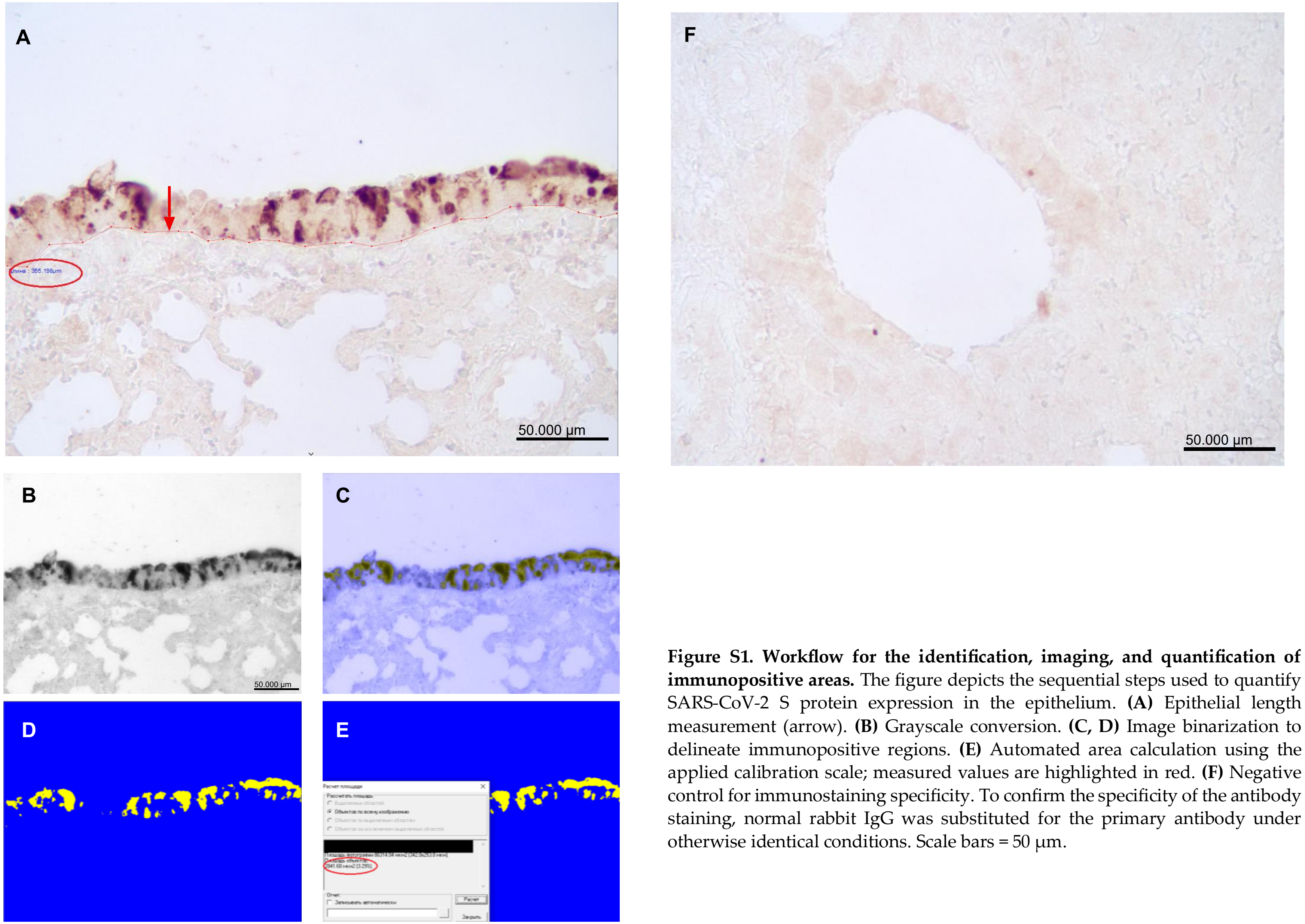

Figure S1. Workflow for the identification, imaging, and quantification of immunopositive areas. The figure depicts the sequential steps used to quantify SARS-CoV-2 S protein expression in the epithelium. **(A)** Epithelial length measurement (arrow). **(B)** Grayscale conversion. **(C, D)** Image binarization to delineate immunopositive regions. **(E)** Automated area calculation using the applied calibration scale; measured values are highlighted in red. **(F)** Negative control for immunostaining specificity. To confirm the specificity of the antibody staining, normal rabbit IgG was substituted for the primary antibody under otherwise identical conditions. Scale bars = 50 µm.

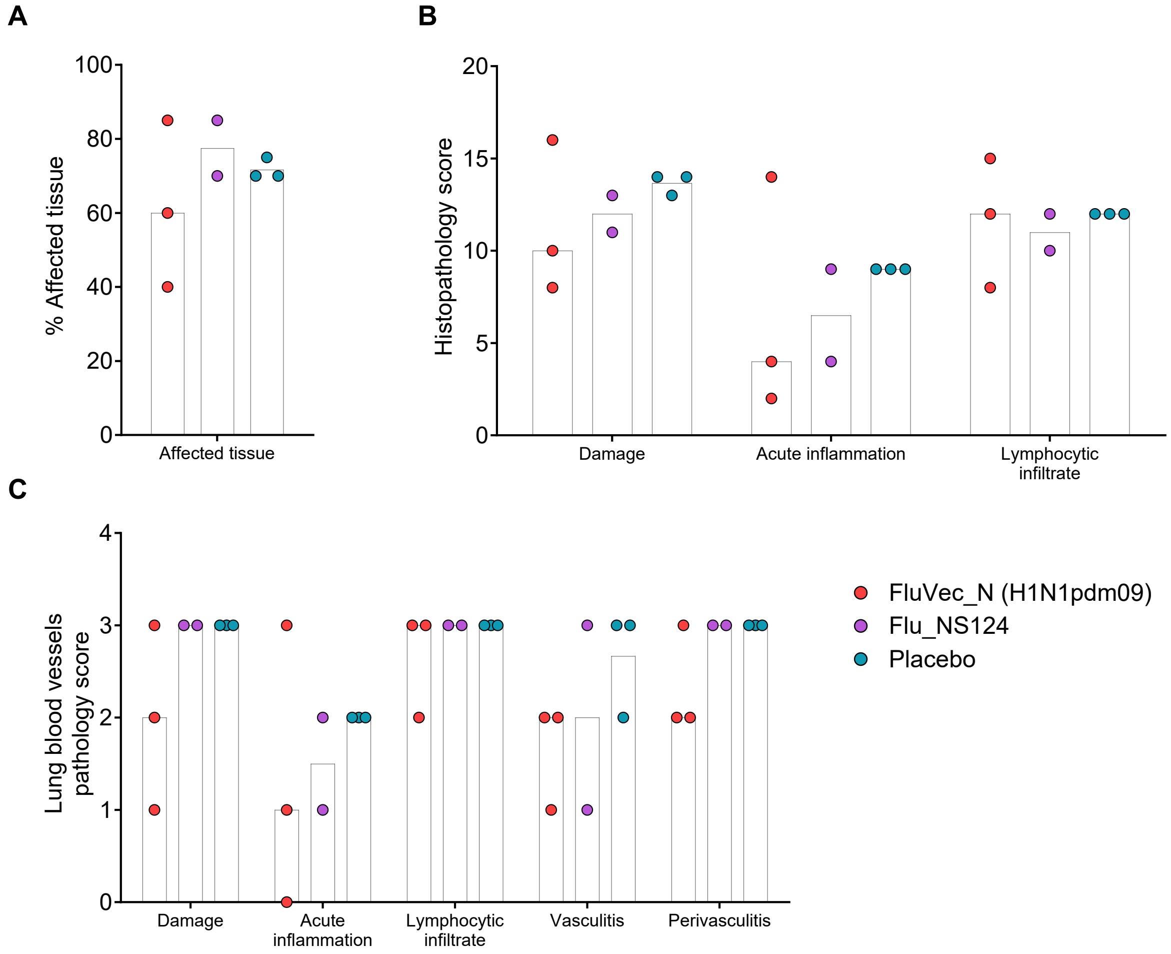

Figure S2. Semi-quantitative assessment of the severity of lung lesions in FluVec_N (H1N1pdm) immunized hamsters following SARS-CoV-2 infection. The proportion of affected tissue (A), overall pathology severity score (B) and lung blood vessels pathology score(C) are shown in the lungs on day 6 after infection.

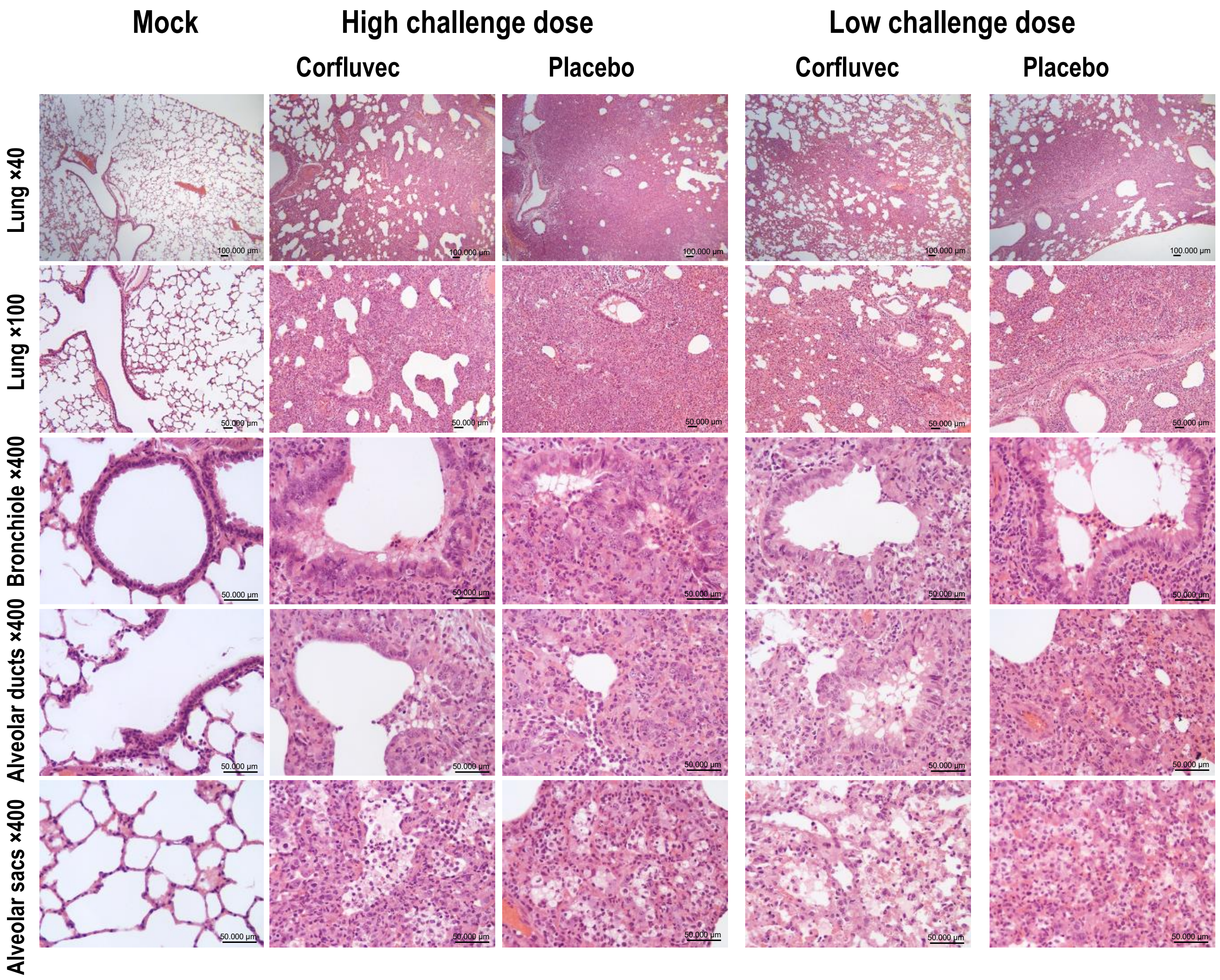

Figure S3. Microscopic structure of the lungs of *Corfluvec* immunized hamsters following SARS-CoV-2 infection. Representative microphotographs showing the most pronounced changes are presented. Day 6 post-infection, hematoxylin and eosin staining. Magnification × 40 (scale bars = 100 µm); magnification× 100 and × 400 (scale bars = 50 µm).

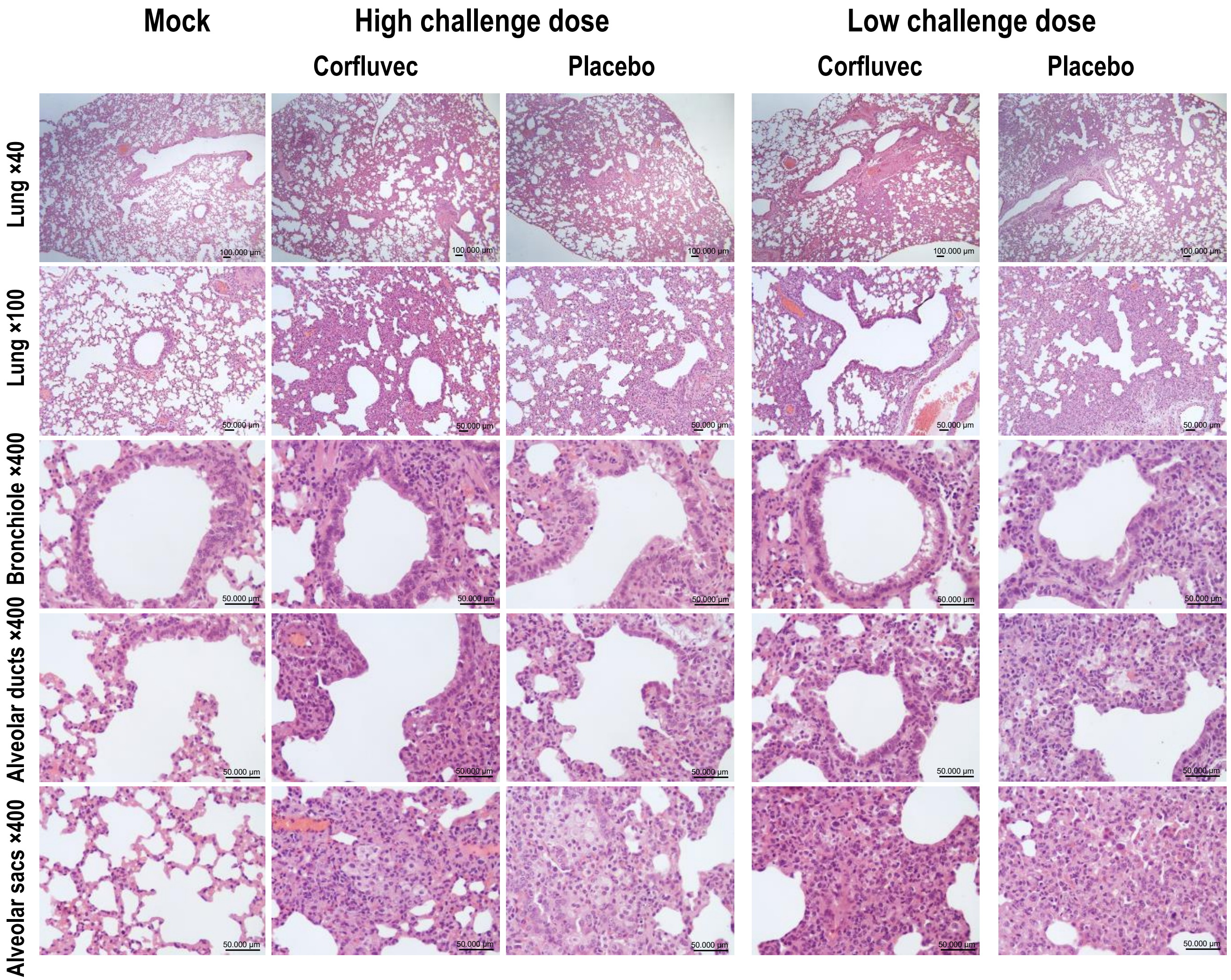

Figure S4. Microscopic structure of the lungs of *Corfluvec* immunized hamsters following SARS-CoV-2 infection. Representative microphotographs showing the most pronounced changes are presented. Day 10 post-infection, hematoxylin and eosin staining. Magnification × 40 (scale bars = 100 μm); magnification× 100 and × 400 (scale bars = 50 μm).

Table S2 – Body weight of cynomolgus macaques, individual readings

Group	No. of animal	Sex	Body weight, g						
			Days						
			-3	1	5	8	21	25	42
Placebo	1.1	♂	7000	6940	6960	6900	6920	6880	7000
	1.2	♀	3600	3380	3560	3600	3480	3520	3500
Corfluvec	2.1	♂	8800	8820	8760	8860	8960	8900	9120
	2.2	♂	6300	6300	6340	6400	6480	6500	6520
	2.3	♂	4500	4520	4540	4560	4580	4540	4540
	2.4	♂	6600	6140	6160	6180	6260	6200	6420

Body weight remained within the physiological range for the species (Supplementary Table S1): females, 2.5–5.7 kg (adult, sexually mature), males, 4.7–8.3 kg (adult, sexually mature). Minor fluctuations observed were associated with short-term fasting prior to anesthesia.

Table S3 – Body temperature of cynomolgus macaques, individual readings measured at three sites (forehead, nose, rectal).

Group	No. of animal	Sex	Body temperature (forehead), °C															
			Days															
			-3	1	2	3	4	5	6	7	21	22	23	24	25	26	27	28
Placebo	1.1	♂	39.2	38.7	38.6	39.2	38.9	38.7	38.4	39.1	39.1	39.6	39.5	39.5	38.9	38.8	39.0	39.2
	1.2	♀	38.4	39.1	38.7	38.5	39.4	38.8	38.7	39.3	39.9	39.6	39.1	38.8	39.2	39.3	38.7	39.4
Corfluvec	2.1	♂	38.6	38.3	38.6	39.2	37.9	39.2	39.4	39.4	38.8	39.4	39.5	39.3	39.4	38.7	38.8	38.6
	2.2	♂	39.1	39.1	38.6	38.7	38.9	38.9	39.1	38.8	38.9	39.5	38.7	39.3	39.3	39.0	39.7	38.7
	2.3	♂	38.8	38.6	39.0	39.4	38.9	39.2	38.6	39.2	39.5	39.5	39.4	39.3	39.5	39.1	39.3	38.9
	2.4	♂	39.3	38.4	38.9	39.4	38.8	38.7	39.0	39.8	39.4	38.9	39.2	38.9	36.9	39.1	39.4	39.0
	M±SEM		39.0± 0.16	38.6± 0.18	38.8 0.10	39.2± 0.17	38.6± 0.21	39.0± 0.12	39.0± 0.17	39.3± 0.21	39.2± 0.18	39.3± 0.14	39.2± 0.18	39.2± 0.10	38.8± 0.06	39.0± 0.09	39.3± 0.19	38.8± 0.09

Group	No. of animal	Sex	Body temperature (nose), °C															
			Days															
			-3	1	2	3	4	5	6	7	21	22	23	24	25	26	27	28
Placebo	1.1	♂	39.3	39.1	39.4	39.6	39.8	39.4	39.6	38.8	39.2	38.8	38.7	39.0	39.4	39.5	39.4	38.8
	1.2	♀	38.6	38.6	38.8	39.2	39.3	38.7	39.1	39.0	38.5	38.9	39.3	39.4	38.6	39.2	38.6	39.1
Corfluvec	2.1	♂	39.2	39.6	39.1	38.9	39.4	39.2	39.2	38.7	39.4	38.4	38.6	38.7	39.0	38.6	39.0	38.9
	2.2	♂	38.9	38.5	38.7	38.6	39.2	38.3	38.5	38.5	39.2	39.0	39.5	39.0	38.7	38.7	38.7	39.0
	2.3	♂	38.4	39.2	39.4	39.3	39.0	39.4	39.3	38.7	38.8	38.9	38.7	38.7	38.6	39.0	38.9	39.2
	2.4	♂	38.6	39.1	38.8	39.0	39.4	38.8	39.1	39.2	39.6	38.8	39.4	39.1	38.8	38.6	38.6	39.5
	M±SEM		38.8± 0.18	39.1± 0.23	39.0 0.16	39.0± 0.14	39.3± 0.10	38.9± 0.24	39.0± 0.18	38.8± 0.15	39.3± 0.17	38.8± 0.13	39.1± 0.23	38.9± 0.10	38.8± 0.09	38.7± 0.09	38.8± 0.09	39.2± 0.13

Group	No. of animal	Sex	Body temperature (rectal), °C															
			Days															
			-3	1	2	3	4	5	6	7	21	22	23	24	25	26	27	28
Placebo	1.1	♂	39.2	38.1	-[a]	38.4	-[a]	38.5	-[a]	-[a]	38.2	-[a]	38.0	-[a]	37.4	-[a]	-[a]	37.9
	1.2	♀	38.1	38.1	-[a]	38.0	-[a]	37.8	-[a]	-[a]	38.5	-[a]	38.0	-[a]	37.6	-[a]	-[a]	37.8
Corfluvec	2.1	♂	38.4	38.4	-[a]	38.1	-[a]	38.3	-[a]	-[a]	38.1	-[a]	37.5	-[a]	38.0	-[a]	-[a]	37.5
	2.2	♂	38.1	38.1	-[a]	38.7	-[a]	38.8	-[a]	-[a]	38.3	-[a]	38.1	-[a]	38.4	-[a]	-[a]	37.3
	2.3	♂	38.8	37.4	-[a]	37.7	-[a]	37.9	-[a]	-[a]	37.7	-[a]	37.5	-[a]	38.2	-[a]	-[a]	37.0
	2.4	♂	39.2	38.6	-[a]	38.5	-[a]	37.7	-[a]	-[a]	37.5	-[a]	37.6	-[a]	38.1	-[a]	-[a]	37.7
	M±SEM		38.6±0.24	38.1±0.26	-[a]	38.3±0.22	-[a]	38.2±0.24	-[a]	-[a]	37.9±0.18	-[a]	37.7±0.14	-[a]	38.2±0.09	-[a]	-[a]	37.4±0.15

-[a]- *No measurement was taken, as the animal was not scheduled for anesthesia on that day*.

Recorded values showed only minor deviations from the normal range for cynomolgus macaques (38.7–39.8°C; source: https://www.nc3rs.org.uk/macaques/welfare-assessment/physiological-indicators/) and were not considered clinically significant. Slightly lower rectal temperatures were likely attributable to the effects of anesthesia.

Table S4 – Haematological parameters of cynomolgus macaques, individual values

Group	Days	No. of animal	RBC, 10^{12}/L	HGB, g/L	HCT, %	PLT, 10^9/L	WBC, 10^9/L	LYM, %	MON, %	GRA, %	MCH, pg	MCV, fL	MCHC, g/L	MPV, fL
Placebo	-3	1.1	5.06	108	35.6	219	8.4	21.6	28.3	50.1	21.3	70.4	303	11.2
		1.2	4.71	104	34.1	520	11.5	56.1	12.8	31.1	22.1	72.4	305	9.5
	7	1.1	4.75	102	33.7	341	12.5	12.4	7.5	80.1	21.5	70.9	303	10.5
		1.2	4.70	103	33.8	538	12.1	52.3	10.2	37.5	23.1	71.2	308	10.3
	20	1.1	5.17	113	36.3	273	6.7	23.4	9.4	67.2	21.9	70.2	311	10.9
		1.2	4.74	110	34.0	532	9.5	48.4	8.3	43.3	22.3	72.2	315	9.1
	27	1.1	5.01	112	34.8	353	8.1	18.4	8.7	72.9	22.4	69.6	322	10.3
		1.2	4.66	113	33.9	609	8.7	49.4	9.6	41.0	24.2	72.7	333	8.4
	42	1.1	5.06	110	34.2	318	9.7	15.0	4.8	80.2	21.7	67.6	322	11.0
		1.2	4.73	110	34.1	569	12.2	29.5	8.5	62.0	23.3	72.1	323	9.0
Corfluvec	-3	2.1	4.85	113	36.7	448	8.1	14.7	6.2	79.1	23.3	75.7	308	7.6
		2.2	5.4	115	37.3	416	10.1	39.3	11.1	49.6	21.3	69.1	308	8.6
		2.3	5.24	94	32.9	272	15	16.3	19.2	64.5	17.9	62.8	286	10.0
		2.4	5.15	115	37.6	422	9.1	25.1	11.2	63.7	22.3	73	306	9.1
	7	2.1	4.89	114	37.4	516	13.1	14.4	6.2	79.4	23.3	76.5	305	7.8
		2.2	5.13	109	36.1	503	9.3	30.6	13.8	55.6	21.2	70.4	302	8.3
		2.3	5.24	98	33.7	390	9.1	41.8	17.0	41.2	18.7	64.3	291	9.2
		2.4	5.04	117	37.4	430	10.6	31.4	8.6	60.0	23.2	74.2	313	9.3
	20	2.1	5.04	121	38.4	502	15.1	9.1	7.2	83.7	24.0	76.2	315	7.2
		2.2	5.05	112	35.4	459	12.5	19.0	11.5	69.5	22.2	70.1	316	8.0
		2.3	5.39	104	34.1	306	11.0	33.1	13.3	53.6	19.3	63.3	305	9.1
		2.4	5.10	123	38.0	397	13.4	25.7	9.1	65.2	24.1	74.5	324	9.6
	27	2.1	4.93	122	37.4	561	11.9	19.2	8.2	72.6	24.7	75.9	326	7.3
		2.2	5.05	116	35.4	432	14.9	34.8	11.7	53.5	23	70.1	328	9.3
		2.3	5.06	99	32.0	183	15.4	51.0	14.4	34.6	19.6	63.2	309	10
		2.4	5.31	132	39.8	500	13.6	35.8	9.5	54.7	24.9	75.0	332	9.1

Group	Days	No. of animal	RBC, 10^{12}/L	HGB, g/L	HCT, %	PLT, 10^9/L	WBC, 10^9/L	LYM, %	MON, %	GRA, %	MCH, pg	MCV, fL	MCHC, g/L	MPV, fL
	42	2.1	5.11	123	37.9	609	11.2	18.1	8.6	73.3	24.1	74.2	325	7.1
		2.2	5.34	119	36.5	471	8.9	24.5	8.9	66.6	22.3	68.4	326	8.4
		2.3	5.47	104	33.4	249	14.1	26.2	8.0	65.8	19.0	61.1	311	9.8
		2.4	5.32	126	38.6	471	6.7	32.9	6.7	60.4	23.7	72.6	326	8.5

Abbreviations: RBC, red blood cells (×10^{12}/L); HGB, hemoglobin (g/L); HCT, hematocrit (%); PLT, platelets (×10^9/L); WBC, white blood cells (×10^9/L); LYM, lymphocytes (%); MON, monocytes (%); GRA, granulocytes (%); MCH, mean corpuscular hemoglobin (pg); MCV, mean corpuscular volume (fL); MCHC, mean corpuscular hemoglobin concentration (g/L); MPV, mean platelet volume (fL).

Table S5 – Biochemical parameters of cynomolgus macaques, individual values

Group	Days	No. of animal	ALB, g/L	ALT, U/L	AST, U/L	CHOL, mmol/L	CREA, µmol/L	GLU, mmol/L	TP, g/L	TG, mmol/L	BUN, mmol/L	BIL-T, µmol/L
Placebo	-3	1.1	34.2	35.5	31.4	3.22	124.9	2.80	66.3	0.71	4.68	1.07
		1.2	30.1	35.5	41.2	3.19	68.2	2.54	59.3	0.98	2.69	0.77
	7	1.1	35.0	73.6	45.3	3.67	107.4	7.64	69.5	0.42	4.82	2.88
		1.2	33.4	65.4	40.1	3.82	71.6	6.33	62.7	0.96	2.54	0.78
	20	1.1	36.7	39.1	32.2	3.68	133.4	5.66	67.7	0.45	3.51	1.34
		1.2	37.2	48.6	28.5	3.76	69.4	5.21	69.2	0.98	2.68	1.35
	27	1.1	35.9	34.5	22.7	4.05	90.8	6.42	72.9	0.48	3.48	1.95
		1.2	33.7	68.6	29.5	3.53	55.2	9.87	66.8	1.17	2.48	1.29
	42	1.1	37.6	32.3	26.6	4.33	111.5	4.35	73.6	0.72	2.60	2.90
		1.2	37.0	46.4	39.3	4.14	72.6	5.65	71.2	0.70	2.85	2.03
Corfluvec	-3	2.1	32.2	34.2	31.0	2.13	140.3	3.67	60.9	0.43	3.86	1.60
		2.2	40.2	19.5	23.0	3.18	105.4	3.61	62.1	0.80	2.27	1.01
		2.3	34.4	29.0	32.4	3.04	102.8	1.16	61.3	0.43	4.75	1.61
		2.4	42.20	24.0	21.5	2.28	86.6	4.04	67.4	0.75	2.62	3.84
	7	2.1	32.3	34.9	26.8	2.16	130.0	4.25	67.2	0.56	3.63	1.83
		2.2	40.9	21.2	17.0	3.73	116.7	5.52	70.9	0.82	5.17	0.96
		2.3	32.8	31.4	25.0	3.28	100.4	4.96	66.5	0.35	3.02	1.67
		2.4	40.30	28.6	18.6	2.63	82.8	5.21	70.7	1.18	2.6	3.18
	20	2.1	36.6	36.0	28.2	2.42	157.3	3.76	67.6	0.65	2.71	2.50
		2.2	44.1	16.2	23.2	3.96	119.2	5.32	72.4	0.64	3.18	6.22
		2.3	36.4	17.0	28.1	2.96	122.2	5.21	68.4	0.20	2.35	1.56
		2.4	44.70	23.6	17.7	2.50	89.7	7.46	67.3	0.90	2.35	2.19
	27	2.1	33.3	28.4	21.8	2.53	112.6	6.23	69.8	1.30	2.1	0.17
		2.2	40.9	24.2	27.3	3.78	88.6	4.55	72.3	0.66	3.31	1.18
		2.3	32.3	30.3	28.1	2.92	81.1	4.96	66.0	0.22	2.49	1.20
		2.4	43.70	27.1	24.6	3.05	57.9	5.12	75.6	2.53	2.84	1.92

Group	Days	No. of animal	ALB, g/L	ALT, U/L	AST, U/L	CHOL, mmol/L	CREA, µmol/L	GLU, mmol/L	TP, g/L	TG, mmol/L	BUN, mmol/L	BIL-T, µmol/L
	42	2.1	36.3	31.4	22.9	2.98	150.9	3.23	75.9	0.46	3.03	4.54
		2.2	41.9	19.0	16.8	3.86	123.7	3.36	79.7	0.36	3.58	2.90
		2.3	32.1	30.0	55.7	3.09	111.0	2.75	69.4	0.33	2.52	3.58
		2.4	44.50	25.3	20.7	3.29	91.0	3.66	72.8	0.64	1.44	4.82

Abbreviations: ALB, albumin (g/L); ALT, alanine aminotransferase (U/L); AST, aspartate aminotransferase (U/L); CHOL, total cholesterol (mmol/L); CREA, creatinine (µmol/L); GLU, glucose (mmol/L); TP, total protein (g/L); TG, triglycerides (mmol/L); BUN, blood urea nitrogen (mmol/L); BIL-T, total bilirubin (µmol/L).
